# Electrochemical and Spectroscopic Characterization of Oxidized Intermediate Forms of Vitamin E

**DOI:** 10.3390/molecules27196194

**Published:** 2022-09-21

**Authors:** Richard D. Webster

**Affiliations:** 1 School of Chemistry, Chemical Engineering and Biotechnology, Nanyang Technological University, 21 Nanyang Link, Singapore 637371, Singapore; webster@ntu.edu.sg; 2 Environmental Chemistry and Materials Centre, Nanyang Environment & Water Research Institute (NEWRI), Nanyang Technological University, 1 Cleantech Loop, Clean Tech One, Singapore 637141, Singapore

**Keywords:** phenols, chromanols, phenolates, cation radicals, phenoxyl radicals, dications, diamagnetic cations, phenoxeniums, *ortho*–quinone methides, hydroxy *para*–quinone hemiketals, *para*-quinones, proton-coupled electron transfer (PCET), single electron transfer (SET), disproportionation, comproportionation, spectroelectrochemistry, bilayer membranes, cyclic voltammetry, chemical oxidation, photochemical oxidation

## Abstract

Vitamin E, a collection of lipophilic phenolic compounds based on chroman-6-ol, has a rich and fascinating oxidative chemistry involving a range of intermediate forms, some of which are proposed to be important in its biological functions. In this review, the available electrochemical and spectroscopic data on these oxidized intermediates are summarized, along with a discussion on how their lifetimes and chemical stability are either typical of similar phenolic and chroman-6-ol derived compounds, or atypical and unique to the specific oxidized isomeric form of vitamin E. The overall electrochemical oxidation mechanism for vitamin E can be summarized as involving the loss of two-electrons and one-proton, although the electron transfer and chemical steps can be controlled to progress along different pathways to prolong the lifetimes of discreet intermediates by modifying the experimental conditions (applied electrochemical potential, aqueous or non-aqueous solvent, and pH). Depending on the environment, the electrochemical reactions can involve single electron transfer (SET), proton-coupled electron transfer (PCET), as well as homogeneous disproportionation and comproportionation steps. The intermediate species produced via chemical or electrochemical oxidation include phenolates, phenol cation radicals, phenoxyl neutral radicals, dications, diamagnetic cations (phenoxeniums) and *para*–quinone methides. The cation radicals of all the tocopherols are atypically long-lived compared to the cation radicals of other phenols, due to their relatively weak acidity. The diamagnetic cation derived from α–tocopherol is exceptionally long-lived compared to the diamagnetic cations from the other β–, γ– and δ–isomers of vitamin E and compared with other phenoxenium cations derived from phenolic compounds. In contrast, the lifetime of the phenoxyl radical derived from α–tocopherol, which is considered to be critical in biological reactions, is typical for what is expected for a compound with its structural features. Over longer times via hydrolysis reactions, hydroxy *para*–quinone hemiketals and quinones can be formed from the oxidized intermediates, which can themselves undergo reduction processes to form intermediate anion radicals and dianions. Methods for generating the oxidized intermediates by chemical, photochemical and electrochemical methods are discussed, along with a summary of how the final products vary depending on the method used for oxidation. Since the intermediates mainly only survive in solution, they are most often monitored using UV-vis spectroscopy, FTIR or Raman spectroscopies, and EPR spectroscopy, with the spectroscopic techniques sometimes combined with fast photoinitiated excitation and time-resolved spectroscopy for detection of short-lived species.

## 1. Introduction

It is the 100-year anniversary of the discovery of vitamin E by Evans and Bishop [1], who in 1922 proposed the existence of an unidentified species that was beneficial in the fertility of rats and aided in preventing the disintegration and assimilation of fetuses in the uterus (known as fetal resorption). The unidentified species was known to be present in certain types of foods such as vegetable oils and lipid-rich plant products, while rats which lacked these components in their diets suffered the fertility problems. Calhoun (a Professor of Greek) and Evans subsequently proposed the name tocopherol for vitamin E as a combination of the Greek words “tocos” meaning childbirth, “phero” meaning to bear, and “-ol” signifying that it is an alcohol [2]. The name vitamin E was given because it was the 5th (after A, B, C and D) essential vitamin micronutrient identified.

**Scheme 1:**
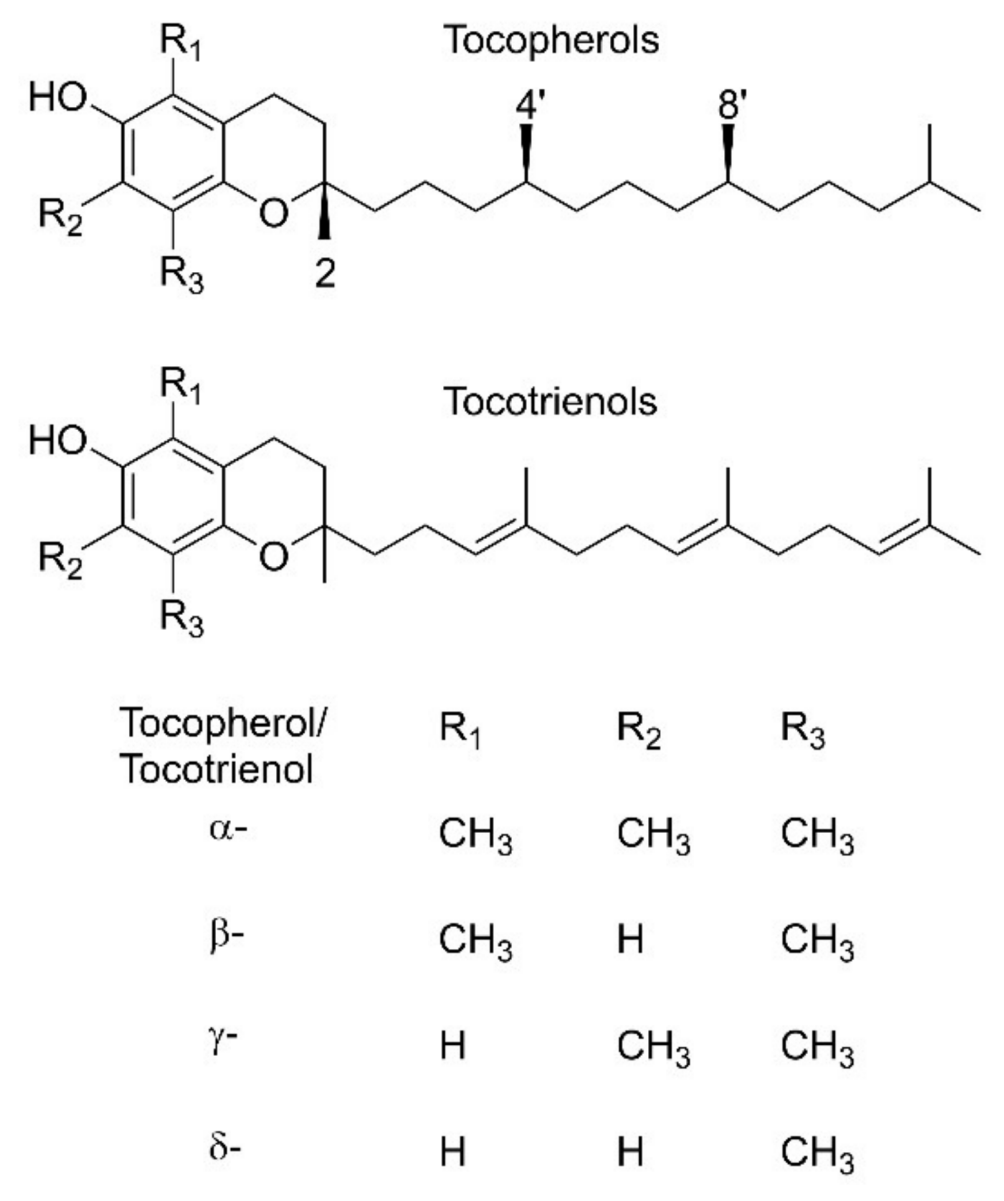
Structures of vitamin E isomers.

Experiments aimed at isolating vitamin E continued into the 1930’s when it was obtained in pure form in 1935 [3], and then in 1938 its structure was solved [4], quickly followed by the first synthesis [5]. Experiments around this time also indicated that vitamin E is comprised of two classes of compound, the tocopherols and tocotrienols, and each of these consist of 4 isomers depending on the degree of methylation of the aromatic phenolic ring of the chroman-6-ol structure, so there are eight major natural forms (Scheme 1) [2,6]. The fully methylated α–tocopherol is the most potent form of vitamin E and is the one that is preferentially taken up and accumulated in human blood cells and tissues [7]. The tocopherols and tocotrienols differ in the side chain, with the tocopherols having a saturated phytyl chain (with maximum hydrogen atoms) and the tocotrienols having multiple double bonds in their hydrocarbon tail resulting in an unsaturated structure. The methyl groups in positions 2, 4′ and 8′ in the tocopherols impart chirality on the phytyl chain. The natural α–tocopherol contains only the fully chiral *RRR*– (formerly designated as D-α–tocopherol) or *2R*– forms, and these are the stereoisomers that are preferentially accumulated in the human body. The synthetic version of α–tocopherol contains equivalent amounts of all the stereoisomers (*SSS*, *SSR*, *SRR*, *SRS*, *RSS*, *RSR*, *RRS*, *RRR*) at the three chiral centers and is designated as the *all racemic* (or *all*–*rac*) α–tocopherol (formerly designated as DL-α–tocopherol). The *all*–*rac*–α–tocopherol and *RRR*–α–tocopherol exist as pale-yellow oils at room temperature and are insoluble in water [8].

Experiments on vitamin E advanced in the 1930’s and 1940’s by Olcott [9] and Mattill [10] and led to the proposal of its abilities as an antioxidant, that is a compound that sacrificially stops lipid peroxidation, and this research has been progressed by many groups until the present day [11], although at various times it has been queried whether vitamin E has another unidentified function in preventing a precise disease or illness, as opposed to the more general antioxidant abilities [11,12,13]. For example, evidence of vitamin E’s role in human childbirth is still lacking compared to the situation in rats [1]. Nevertheless, vitamin E is known to be an essential component of diet with deficiencies leading to various neurological problems, but there are conflicts regarding the reasons for the health effects [14]. Currently, there are two main views on vitamin E’s function is humans, one is as an antioxidant and nothing more [15,16], and the other is having non-antioxidant functions [17], but with both scenarios leading to the same neurological problems. Supporters of the antioxidant function have the advantage that detailed chemical mechanisms have been established about how phenolic compounds act as antioxidants, through hydrogen atom transfer reactions as well as radical coupling reactions [18,19]. The molecular mechanism is most often formulated as vitamin E (TOH) terminating or inhibiting autoxidation cycles by firstly transferring a hydrogen atom to an oxidized lipid site within a cell membrane (LOO^•^) to produce a molecule of lipid hydroperoxide (LOOH) and the vitamin E radical (TO^•^) (Equation (1)). Next, the TO^•^ radical further reacts with another LOO^•^ radical (Equation (2)) in a coupling reaction, resulting in one TOH molecule preventing lipid peroxidation at two sites. In contrast the non-antioxidant functions have no clear chemical mechanism to account for their effects, although they are proposed to involve the same radical intermediate (TO^•^) [20,21,22].

LOO^•^ + TOH → LOOH + TO^•^ (1)

LOO^•^ + TO^•^ → (TO)OOL (2)

A specialized protein called the α–tocopherol transfer protein (α–TPP) resides in the liver and is responsible for delivering α–tocopherol to the very low-density lipoprotein (VLDL) [23,24], which is then transferred and delivered to peripheral cells [7]. The other tocopherols (β, γ and δ) are not retained by α–TPP and instead are metabolized and excreted [25]. Therefore, there must be a reason why α–tocopherol is especially retained at the expense of the other tocopherol isomers. It has been argued that α–TOH is able to react faster with free radicals than the other tocopherols when reactions are carried out in organic solvents, and this is the reason for its preferred uptake [18], although this explanation has been disputed for reactions carried out under micellar-like environments, where the reaction rates are similar for the different isomers [26]. People who have mutations in the α–TPP suffer neurologic abnormalities due to depletion of α–tocopherol in the tissues. Healthy people who have inadequate vitamin E in their diets can have low levels of α–tocopherol in the red blood cells, but in clinical trials this has not led to any evidence of the low-density lipoprotein (LDL) undergoing damage caused by autooxidation [27]. Therefore, it is predominantly people who have genetic defects in the α–TPP that suffer disorders due to vitamin E deficiency, while healthy people show few deleterious effects of low dietary levels despite vitamin E being proposed as the critical molecule in preventing oxidative damage [7]. Furthermore, relatively low daily levels of dietary vitamin E are needed despite the antioxidant mechanism requiring it to act as sacrificial compound [28,29], with this seemingly contradictory observation having been rationalized on the basis that vitamin E can undergo self-replenishment through hydrogen atom transfer reactions with other sacrificial compounds, such as ascorbic acid [30].

An interesting feature of vitamin E that is seldom discussed with respect to the antioxidant mechanism is the exact location that it resides within the cells [8]. The compound is lipophilic due to the long alkyl hydrocarbon tail, but it also contains the polar hydroxy group in the chroman-6-ol structure (Scheme 1). It is generally thought that vitamin E can arrange itself within the lipid bilayers with the hydrocarbon tail pointing into the lipid bilayer, and the hydrophilic phenolic head orientated closer to the aqueous interface [8,31,32,33]. However, the antioxidant mechanism prefers that the compound is able to move freely within the fluidic like membrane so that it is able to react at oxidized sites on lipid cell walls, rather than being confined in one location [34]. Most of the biochemical experiments on vitamin E do not consider its exact position within the lipid bilayer when accounting for the antioxidant properties.

There are several important features relating to the exact structure and properties of α–tocopherol that are difficult to understand based on its proposed function solely as an antioxidant. These unusual features include: (i) Why is the entire chroman-6-ol structure important when it is only the phenolic group that undergoes the hydrogen atom transfer reaction? (ii) Why is α–tocopherol preferred over the other tocopherols when all undergo the same reaction? Specifically, why does the body exclude the other tocopherols when it could be considered that more is better to prevent lipid peroxidation? (iii) Why does the body specifically want the chiral *RRR*–form of α–tocopherol? The chirality of the phytyl tail will impart a more rigid geometry, but this would only be important if the compound were locked in a specific position which is difficult to rationalize based on the antioxidant mechanism. It has been noted that there are inconsistencies and lack of uniformity in the form of vitamin E that is administered between clinical trials, with most studies conducted with *all*–*rac*–α–tocopherol rather than *RRR*–α–tocopherol, and sometimes the exact stereoisomeric form is not even reported [35]. This has led to uncertainties in the precise daily dosage of vitamin E that is needed along with questions on the reliability of conclusions from neurological studies based on experiments conducted with *all*–*rac*–α–tocopherol [35,36].

To date, nearly all the biological chemistry of vitamin E has been linked to two major forms; the phenol starting material and its associated phenoxyl radical formed when it transfers a hydrogen atom to sacrificially limit lipid peroxidation. Nevertheless, it will be shown in this review that there are several other oxidized forms of vitamin E that have very interesting properties, some of which do require both the chroman-6-ol structure as well as the fully methylated α–form to increase their lifetimes in solution and enable them to be reversibly reduced back to the starting material. Therefore, the aim of this review is to highlight other oxidized forms of vitamin E that might be important in non-antioxidant mechanisms, to summarize their characteristic electrochemical and spectroscopic properties, and to comment on how the oxidized forms of vitamin E are similar or different to non-vitamin E compounds but of a similar class (phenols and chromanols). In situ spectroscopic characterization is especially important because many of the intermediates are short-lived and therefore, need to be detected in the same environment that they are generated. Modern advances in spectroscopic techniques enables more in situ measurements to be made, and it may be possible in future to test for the existence of these intermediate oxidized compounds under in vivo conditions.

## 2. Preparation of Oxidized Compounds through Chemical, Photochemical, and Electrochemical Processes

**Scheme 2:**
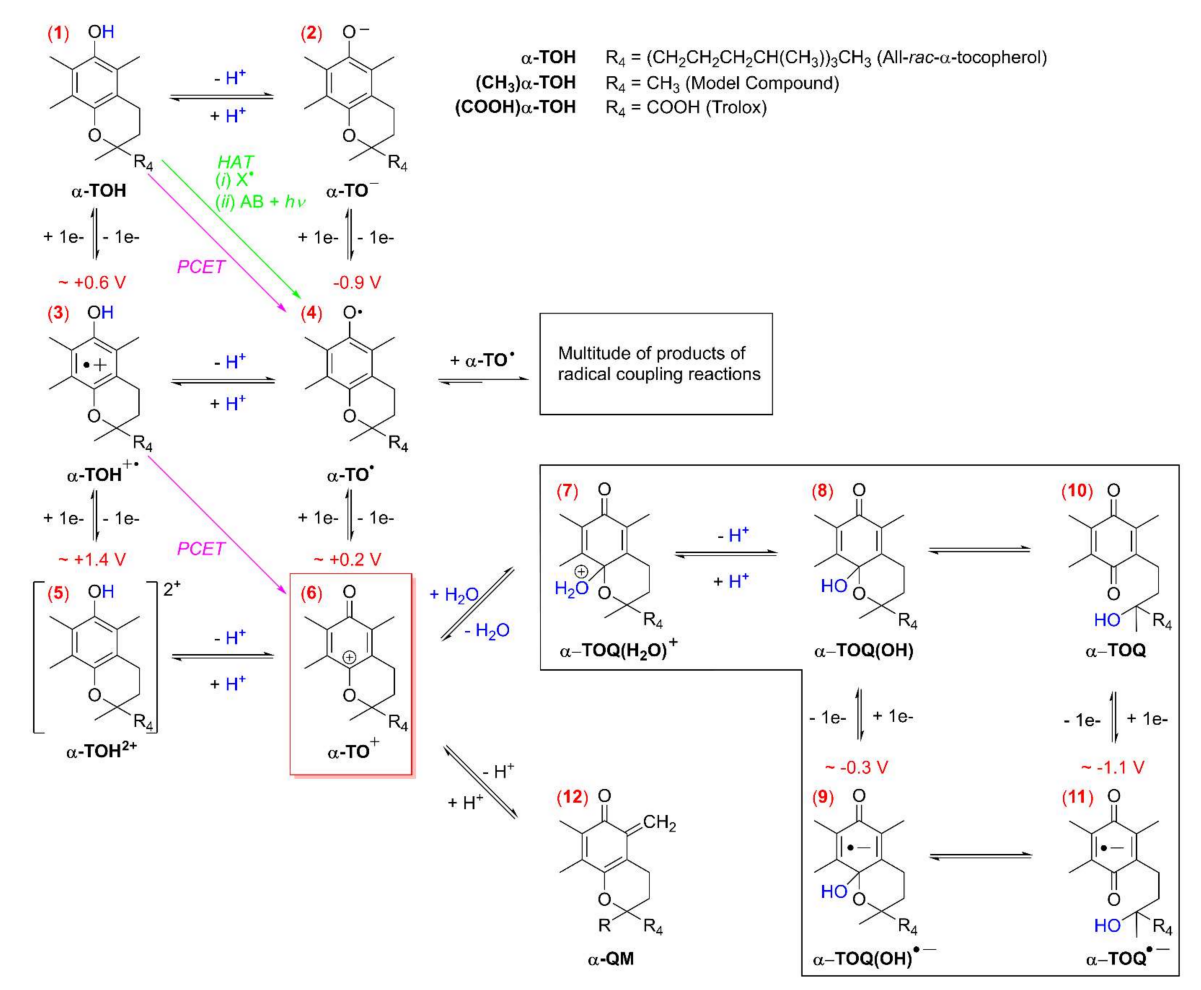
Oxidation mechanism of α–tocopherol showing major intermediate forms and stable products. HAT = Hydrogen atom transfer. PCET = Proton–coupled electron transfer.

This review primarily discusses intermediate species that are produced via the oxidation of vitamin E, rather than the long-term oxidation products that are usually unable to undergo conversion back the phenolic starting material. Scheme 2 shows that main intermediate oxidation products that can be formed through either chemical, photochemical, electrochemical or a combination of mechanisms. The mechanism is illustrated for the α–tocopherol form of vitamin E although the reactions are applicable for most of the tocopherols and tocotrienols (except for compound **12** in Scheme 2 that requires a methyl group adjacent to the phenol group for its formation), with the differences between the different isomers primarily related to the rates of the individual steps and the lifetimes of the species in solution. Similarly, the aliphatic side group (R_4_ in Scheme 2) does not affect the reactions is Scheme 2, other than altering the rates for some of the transformations and the lifetimes in solution of some of the species.

The three major R_4_ groups on compounds that are used in oxidation experiments are the racemic phytyl tail in *all*–*rac*–α–tocopherol (hereafter termed α–TOH), a carboxylate group in the water-soluble derivative named Trolox [(COOH)α–TOH], and a methyl group in the derivative abbreviated (CH_3_)α–TOH (Scheme 2). For in vivo studies, the tocopherols containing the full aliphatic chain need to be used to ensure that the lipophilic compound is taken up into the VLDL and other peripheral cells. The *all*–*rac*–α–tocopherol (or other isomers) are often used for biological studies rather than *RRR*–α–tocopherol because the racemic mixture is considerably less expensive than the fully chiral form. For ex situ studies examining oxidation products, α–TOH is most often used, along with the methyl substituted derivative [(CH_3_)α–TOH]. Experiments with (CH_3_)α–TOH have the advantages that the compound can be easily synthesized in large amounts in very high purity (>99.99%) [37], the compound is a solid at room temperature and so easily handled, it remains stable in air at room temperature for long periods (~years), and without the long aliphatic chain, the oxidation products can be simpler to purify and identify by spectroscopic techniques.

An interesting feature associated with the proposed antioxidant function of vitamin E is that many natural metabolic reaction products do not mimic the final products found in ex situ oxidation experiments. The major products identified from the metabolic reactions of α–TOH are species where the aliphatic chain has undergone oxidation whilst the chroman-6-ol ring structure remains [38]. In contrast, experiments using chemical or photochemical oxidation pathways aimed at preparing the α–TO^•^ radical involve chemical changes to the chroman-6-ol structure, often producing higher molecular weight compounds through radical coupling pathways [39]. Therefore, uncertainties exist whether non-natural methods for initiating oxidation of vitamin E to study its potential as a radical scavenger, results in the same reaction pathways that occur in entirely natural systems. Another factor for consideration when studying potential chemical mechanisms that α–TOH may undergo in biological systems, is that detected metabolic products may not be significant in its biological function if α–TOH was undergoing completely reversible chemical transformations, such that the chroman-6-ol structure is always retained. This has already been proposed for α–TO^•^ where other hydrogen atom donors may be able to regenerate α–TOH [30], but there are further oxidized intermediate forms that could also undergo reversible transformations involving electrons and protons, as indicated in Scheme 2, which are not currently linked to vitamin E’s biological function(s). Finally, experiments aimed at producing oxidized forms of vitamin E by forcing transformations through unnatural means (such as light driven processes) or under extreme conditions (such as high concentrations) may not be relevant to understanding its true biological properties, since the compound does not experience the same conditions in vivo.

### 2.1. Hydrogen Atom Transfer (HAT) Reactions

Most oxidation experiments performed on α–TOH have been designed to generate the α–TO^•^ radical to study its reactivity and chemical transformations [39,40,41,42]. It can be argued that one of the reasons for the large number of reports on the α–TO^•^ radical and its properties is that very good tools are in place to initiate and monitor its reactions under in vitro or even in vivo conditions. Pulse radiolysis enables both the generation of the radical by a rapid photochemical reaction and a simultaneous detection by measuring its UV-vis absorption spectrum [40]. EPR spectroscopy enables monitoring of the radical signal and has the advantage that background signals are almost completely absent since only paramagnetic species are observed, thus enabling the reactions to be monitored in highly complex and inhomogeneous media [41].

**Scheme 3:**
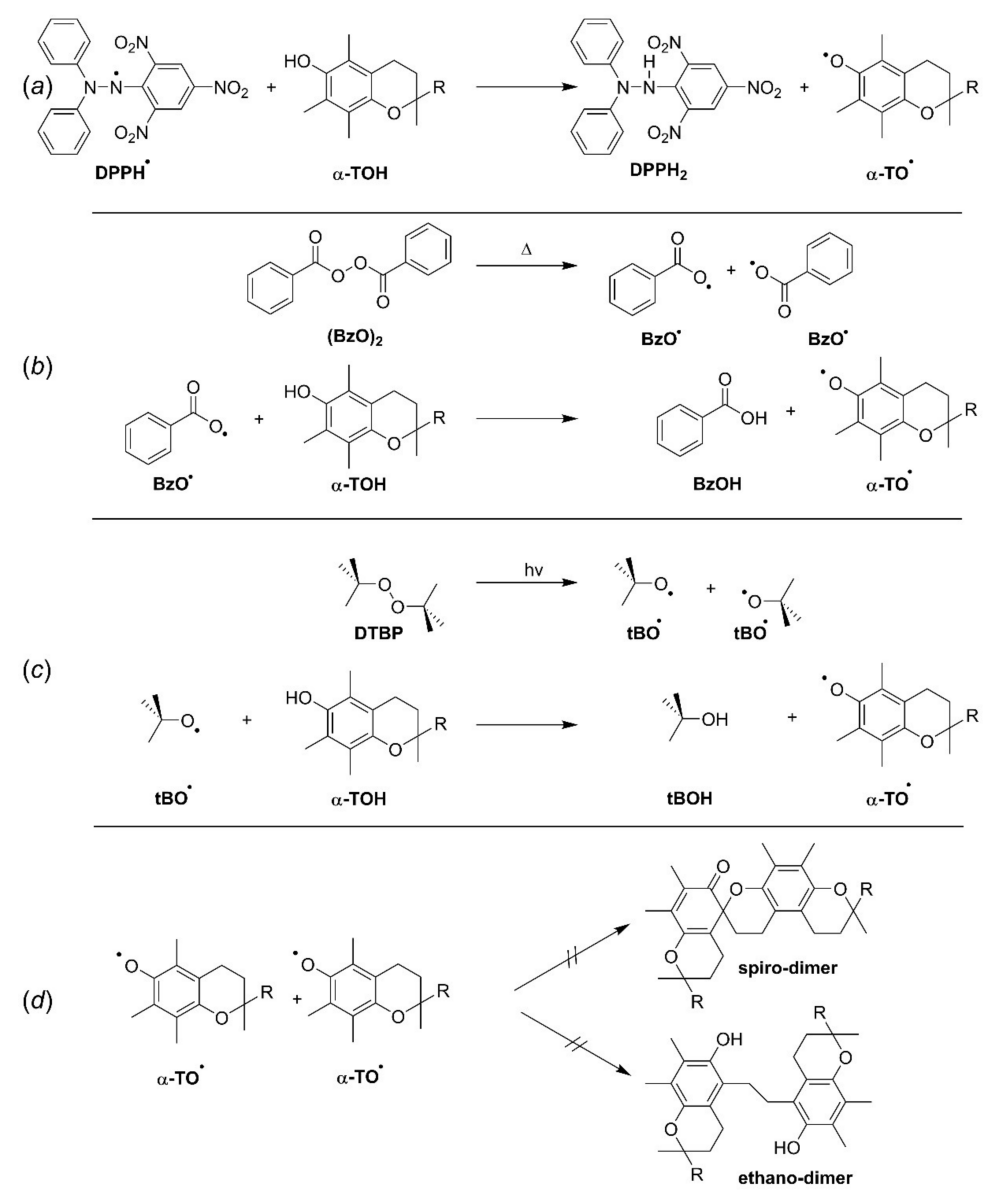
Methods used to produce α–TO^•^ from hydrogen atom transfer (HAT) reactions. (**a**) Reaction with DPPH; (**b**) Reaction with BzO^•^ formed from (BzO)_2_, (**c**) Reaction with tBO^•^ formed photochemically from DTBP. (**d**) Dimer reaction products of α–TO^•^.

The simplest pathway for generating α–TO^•^ from α–TOH is via a HAT reaction as shown in the green diagonal line in Scheme 2, which can be conducted experimentally by (i) adding stable or semi-stable hydrogen atom abstraction agents, or (ii) photochemically dissociating a compound into a neutral radical that then abstracts the phenolic hydrogen atom from α–TOH. An example of a stable hydrogen atom abstraction agent is 2,2-diphenyl-1-picrylhydrazyl (DPPH^•^), which converts to DPPH_2_ [Scheme 3a] [41]. An example of a semi-stable radical initiator is benzoyl peroxide [(BzO)_2_] which is thermally unstable and dissociates into two benzoyloxy radicals (BzO^•^) [Scheme 3b] [42]. An example of a photochemically active compound is di–*tert*–butyl peroxide (DTBP) which dissociates under UV-vis light into two *tert*–butoxyl radicals (tBO^•^) [Scheme 3c] [40]. Once formed, α–TO^•^ quickly converts into other usually higher molecular weight compounds, including the ethano-dimer and the spiro-dimer (likely via an *ortho*–quinone methide intermediate [42]) which are shown in Scheme 3d [39,40,41,42,43].

A large range of compounds are known to form via the chemical or photochemical reactions with the exact identities and yields depending on the experimental parameters, including the solvent and reagents used as well as the temperature and concentrations of reactants. However, since these are stable compounds that cannot be easily converted back to the phenolic starting material, they are not discussed further in this review. The stable long term dimeric oxidation products have not been shown to have any biological function and they may also not form under natural oxidation conditions, since α–TO^•^ is not long-lived and will rapidly react with nearby lipophilic molecules.

### 2.2. Electrochemical Oxidation

There are considerably fewer mechanistic studies that use electrochemical methods to oxidize vitamin E, compared to the chemical and photochemical methods. However, one advantage of voltammetry is that it allows the generation of oxidized forms via electron transfer to an electrode surface whilst simultaneously probing the fate of the oxidized species by measuring the current that flows. Furthermore, varying that voltammetric scan rate allows information on the rates of reaction of intermediate oxidized species, and therefore, their lifetimes in solution. The drawback of electrochemical methods is that they require specific solvents that have high dielectric constants as well as the addition of large quantities of supporting electrolyte to reduce the solution resistance to enable the optimal voltammetric response. An interesting question about electrochemistry is whether it is relevant to biological reactions since in vivo electron transfer reactions do not occur at solid electrode surfaces (although analogous criticisms can be applied to the photochemical HAT reactions discussed in Section 2.1). Nevertheless, electrochemical reactions that occur homogeneously in solution via an outer sphere mechanism, that is, direct electron transfer without binding interactions, can be expected to occur at the same potential regardless of whether a solid electrode or chemical redox agent are used. In contrast, for an inner sphere electron transfer reaction which requires direct binding between the electron acceptors and donors, the potential is likely to be significantly shifted [44].

#### 2.2.1. Aqueous Conditions

Although vitamin E is not soluble in pure water, voltametric experiments on α–TOH have been performed in a variety of aqueous environments. These include experiments in aqueous-organic mixtures [45], in organic solvents containing aqueous acids [46], under biphasic conditions at a liquid-liquid interface [47], as oils deposited on electrode surfaces and immersed in aqueous solutions [48,49], and incorporated in lipid multilayers on electrode surfaces and immersed in aqueous solutions of varying acidity [49,50]. Under these aqueous-based environments, the oxidized vitamin E generally undergoes a rapid hydrolysis reaction to form the tocopherol quinone (α–TOQ) as shown in Scheme 2 (compound **10**). However, high water content environments are possibly not relevant to the biological conditions where vitamin E resides within the hydrophobic lipid bilayers, whereas dry aprotic organic solvents may be a closer model to the natural system and allow stabilization of reactive intermediates.

#### 2.2.2. Aprotic Conditions

The earliest voltametric experiments performed on α–TOH in aprotic solvents were by Parker et al. [51,52] and Marcus and Hawley [53] in the late 1960s and beginning of the 1970s. The authors concluded that the compound likely underwent a two-electron oxidation with the loss of a proton and proposed the formation of a diamagnetic cation where the positive charge was situated on either the ether oxygen in the chroman-6-ol ring or the carbon atom adjacent to the oxygen atom in the formed quinoid ring. It was not until the first decade in the 21st century that the system was studied again intensively using voltammetry and spectroscopy by Williams and Webster [54] that more details of the mechanism were determined. Further extensive experiments by Webster’s group over the next decade under a range of conditions led to many more details of the electrochemical mechanisms being unraveled as well as spectroscopic characterization of many oxidized intermediates [49,50,54,55,56,57,58,59,60,61,62,63,64,65,66,67,68]. With hindsight of over 50 years of experiments performed on other phenolic compounds, it became apparent that the electrochemical behavior of vitamin E, particularly the α–TOH form, was remarkably different than what was normally encountered for compounds with a similar structure.

**Figure 1:**
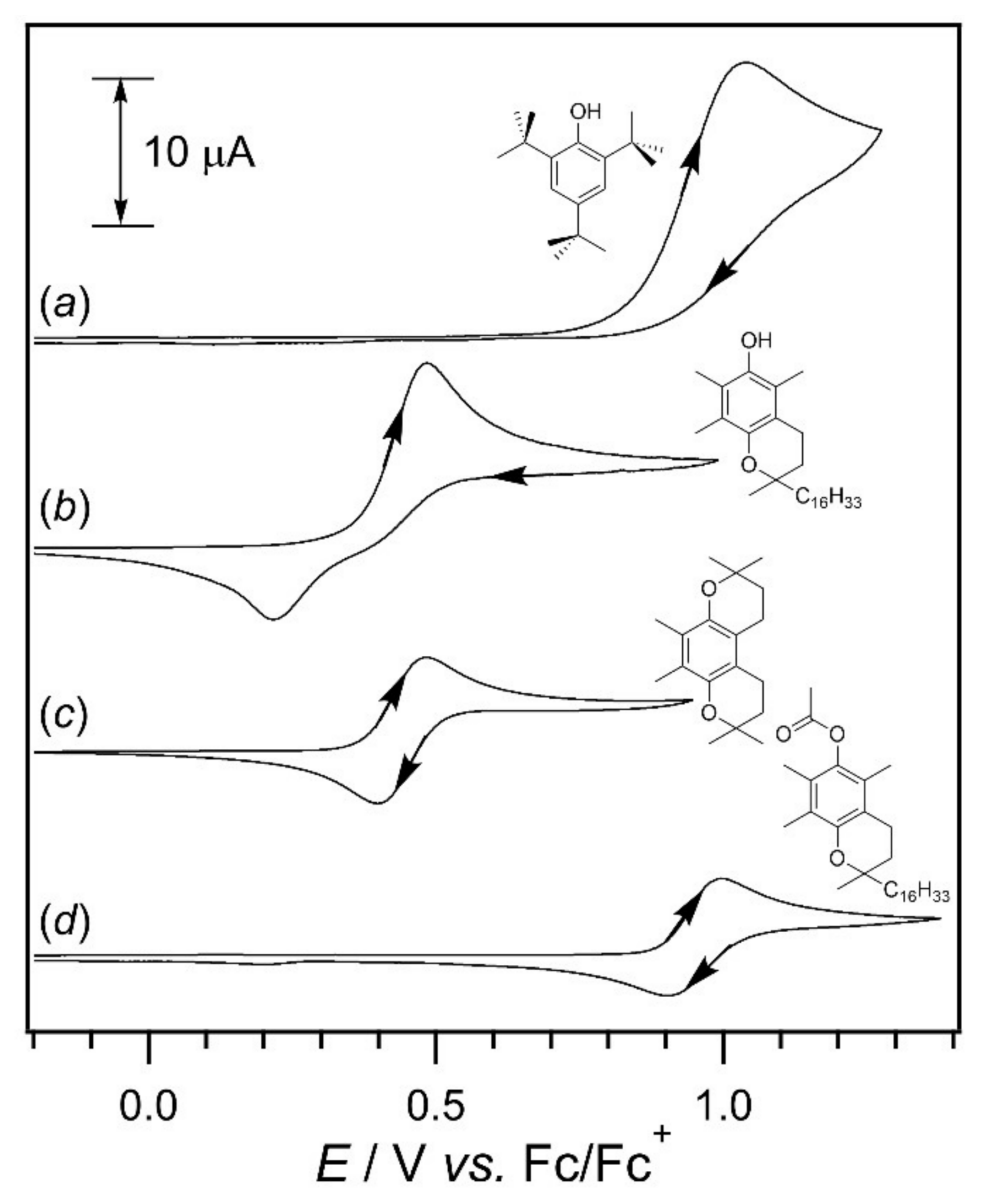
Cyclic voltammograms of ca. 2 mM substrates in CH_3_CN containing 0.25 M *n*–Bu_4_NPF_6_ recorded at 0.1 V s^−1^ scan rate at a 1 mm diameter planar circular Pt electrode at 22 (±2) °C. (**a**) 2,4,6–tri–*tert*–butylphenol [69]. (**b**) α–TOH [56]. (**c**) 3,3,5,6,8,8–Hexamethyl–1,2,3,8,9,10–hexahydropyrano [3,2-f]chromene (HMPC) [61]. (**d**) α–Tocopherol acetate [65].

To understand the unusual voltammetry of vitamin E, it is useful to compare its behavior to other phenolic and chromanol compounds. Figure 1a displays a cyclic voltammogram (CV) of 2,4,6–tri–*tert*–butylphenol that undergoes a two-electron oxidation coupled with one-proton loss (–2e^−^/–1H^+^) to form a diamagnetic cation that is highly reactive in all media (aqueous and non-aqueous) and therefore, cannot undergo conversion back to the starting material when the CV scan direction is reversed, and a reducing potential is then applied [69]. The potential axis in Figure 1, and most subsequent figures that include voltammograms are referenced to the ferrocene^0/+^ redox couple (Fc/Fc^+^), which is the standard internal reference used for voltammetry in non-aqueous solvents [70]. The chemically irreversible feature of the CV of 2,4,6–tri–*tert*–butylphenol is typical of most phenols that form highly reactive oxidized compounds during the forward scan and are unable to be reduced back to the starting material regardless of the scan rate [69,71,72,73,74,75,76]. The rare exception to this is phenols that undergo intramolecular hydrogen bonding of the phenol hydrogen atom with an amine, which can result in stabilization of the initially formed cation radical and allow the appearance of a partially chemically reversible CV [77].

Figure 1b shows the CV of α–TOH that has similarities and differences to CVs of other phenols. The forward scan has been shown to involve the loss of two-electrons and one-proton [52,53,54] but compared to other phenols it is unusual that a reductive peak is observed when the scan direction is reversed after first oxidizing the compound. Controlled potential electrolysis experiments have confirmed that the oxidized compound survives many hours in solution providing that the water content of the solvent is low, and the starting material can be completely regenerated from the oxidized form by applying a reducing potential [54,55]. Therefore, unlike other phenols, α–TOH forms a long-lived diamagnetic cation (compound **6** in Scheme 2) via oxidation (–2e^−^/–1H^+^) and can be converted back to the staring material on the short CV (≤seconds) and long electrolysis (≥min) timescales.

The reason that nearly all phenols undergo a two-electron oxidation on the CV timescale is that the cation radical (compound **3** in Scheme 2) that is formed in the initial one-electron oxidation is very acidic and immediately deprotonates to form the neutral phenoxyl radical (compound **4** in Scheme 2). The phenoxyl radical is easier to reduce than the starting material and so immediately undergoes a further one-electron oxidation to form the diamagnetic cation (compound **6** in Scheme 2). For most phenols, the equivalent diamagnetic cations are highly reactive, whereas for α–TOH, its diamagnetic cation (α–TO^+^) is remarkably very long-lived in the absence of nucleophiles, including trace water.

If the structure of α–TOH is changed so that a labile phenolic hydrogen atom is not present, such as for HMPC [61,78,79,80] and α–tocopherol acetate [65] shown in Figure 1c,d, respectively, then the compounds undergo chemically reversible one-electron oxidation to form long-lived cation radicals. While HMPC is oxidized at the same potential as α–TOH, α–tocopherol acetate is considerably harder to oxidize by approximately 0.5 V. The higher oxidation potential of α–tocopherol acetate compared to α–TOH is the reason that it is frequently used in place of α–TOH in certain formulations (such as skin moisturizers and lotions) because it is more difficult to spontaneously oxidize in the atmosphere and therefore, has a much longer shelf-life, although it can be metabolically converted into α–TOH once it is absorbed through the skin.

#### 2.2.3. Square-Scheme Mechanism and pH Effects

**Scheme 4:**
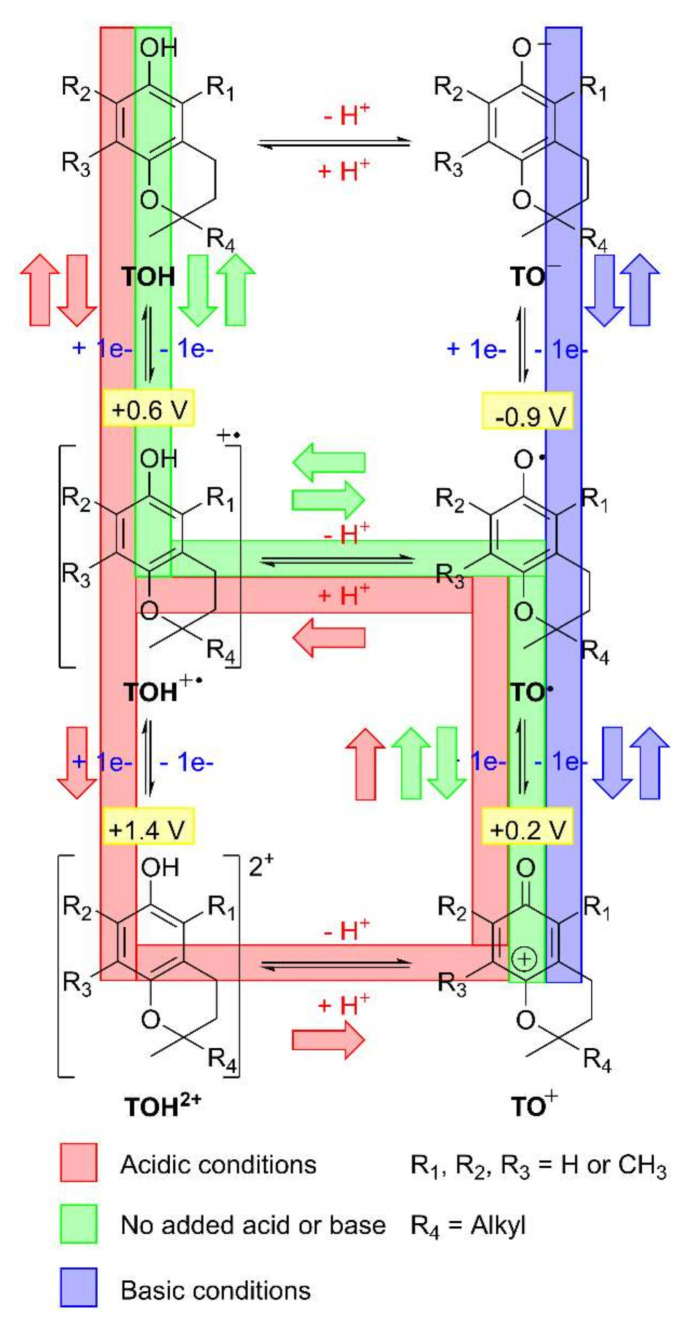
Electrochemical “square–scheme” mechanistic routes for tocopherols and tocotrienols in aprotic organic solvents in the presence and absence of organo-soluble added acids or bases.

**Figure 2:**
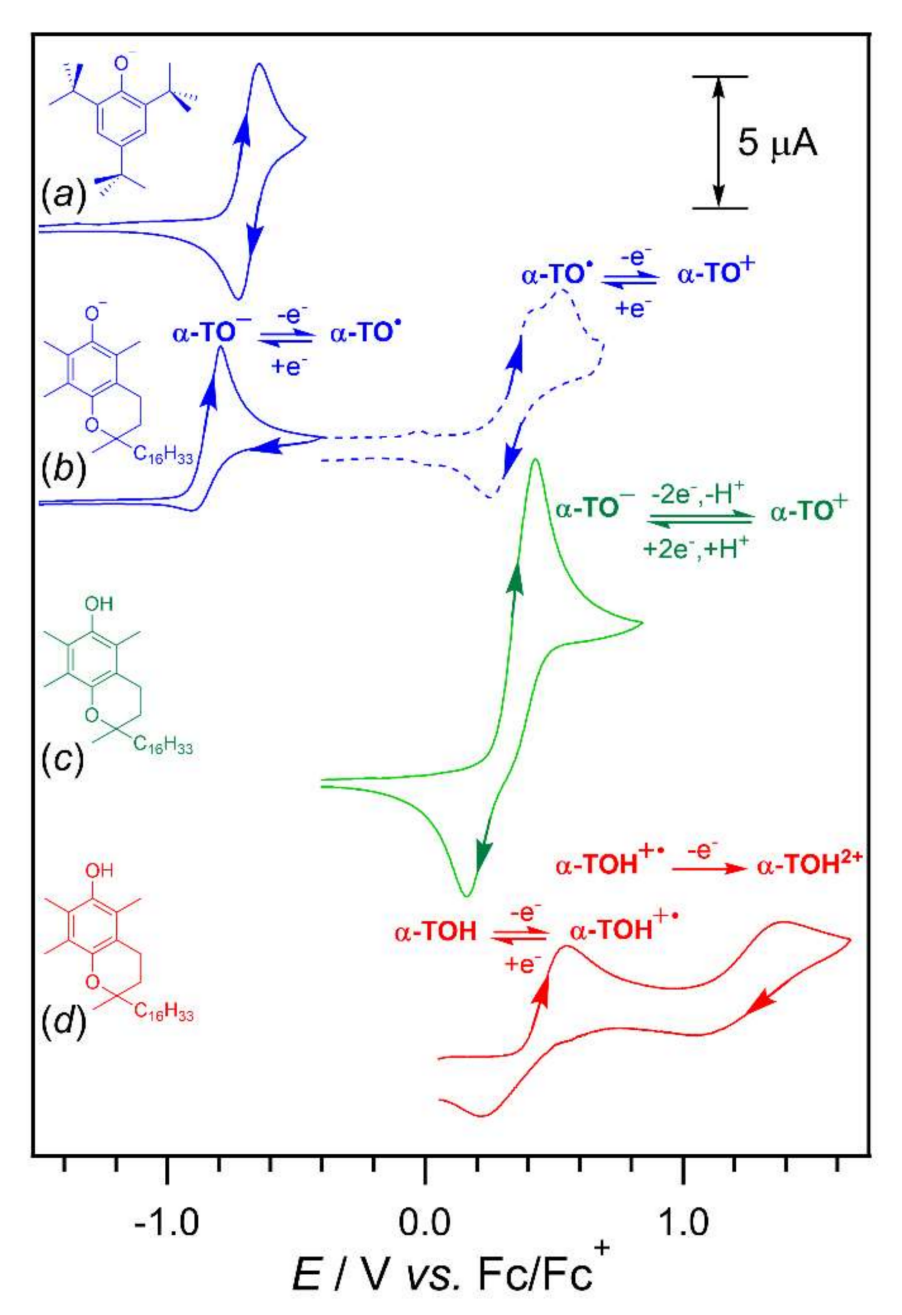
Cyclic voltammograms of ca. 2 mM substrates in CH_3_CN containing 0.25 M *n*–Bu_4_NPF_6_ recorded at 0.1 V s^−1^ scan rate at 1 mm diameter planar circular Pt or glassy carbon electrodes at 22 (±2) °C, under (**a**,**b**) basic, (**c**) approximately neutral, and (**d**) acidic conditions. (**a**,**b**) Phenolates prepared by reacting starting phenols with a molar equivalent of dry Et_4_NPF_6_. (**c**) No added acid or base. (**d**) Addition of 0.1 M neat CF_3_SO_3_H [54].

The “square-scheme” electron and proton transfer reactions on the left-hand side of Scheme 2 can be divided into varying pathways depending on the amount of acid or base present in the aprotic organic solvent, as illustrated in Scheme 4 [56]. When no acid or base is added, the vitamin E compounds (tocopherols and tocotrienols) are oxidized according to the green pathway in Scheme 4. The starting phenolic compound (TOH) is oxidized by one-electron to form the cation radical (TOH^+•^), which then rapidly deprotonates to form the neutral phenoxyl radical (TO^•^). TO^•^ is easier to oxidize than the starting material by several hundred mV so is immediately further oxidized at the electrode surface to form the diamagnetic cation (TO^+^). TO^+^ is also sometimes referred to as a “phenoxenium ion”, and its structure is often drawn with the positive charge residing on one of the oxygen atoms, although spectroscopic data and theoretical calculations indicate that the positive charge is located away from the oxygen atoms [55]. The electrode potentials given in Scheme 4 were estimated by digital simulation techniques and are approximate values (~+/−0.1 V) and can vary depending on the trace water content of the solvent and the exact form (α, β, γ, δ) of the tocopherol and tocotrienol [54,56,58,60,65]. In the absence of added acid or base, the TO^+^ can be reduced back to the starting material by the same green pathway shown in Scheme 4 (the forward and reverse reactions are completely chemically reversible). The green pathway in Scheme 4 is an example of an electrochemical “ECE” mechanism, where “E” signifies an electron transfer and “C” signifies a chemical step, which in this case is a proton transfer. Due to the second electron transfer occurring at a lower (less positive) potential on the forward oxidative scan direction, and occurring at a higher (more positive) potential on the reverse reduction scan, only one peak is detected for the two-electron process in the forward and reverse directions under approximately “neutral conditions” [Figure 2c]. For the purpose of this discussion, the expression “neutral conditions” means an aprotic organic solvent without the addition of acid or base, although the pH was not specifically measured (due to difficulty in estimating the pH in an organic medium).

When a strong organo-soluble base is added to the solution in equal molar amounts, such as tetraethylammonium hydroxide [69,71,72,74,75,76], the phenol starting material is completely deprotonated to form the phenolate anion (TO^−^) [74]. Due to its negative charge, the phenolate is much easier to oxidize than the phenol by approximately 1.5 V, to form the phenoxyl radical by heterogeneous one-electron transfer as shown in the blue pathway in Scheme 4. The blue solid trace in Figure 2b is a CV of the one-electron oxidation of α–TO^−^ at approximately –0.9 V vs. Fc/Fc^+^ to generate α–TO^•^. Because the potential that is applied to generate α–TO^•^ is much lower (less positive) in basic conditions (–0.9 V vs. Fc/Fc^+^) compared to under approximately neutral conditions (+0.6 V vs. Fc/Fc^+^), α–TO^•^ does not undergo immediate further oxidation at the electrode surface. However, the blue solid trace in Figure 2b shows that if the potential direction is reversed after first oxidizing α–TO^−^ by one-electron, only a small peak is detected for the reduction of α–TO^•^ back to α–TO^−^, due to α–TO^•^ being relatively reactive [74]. The reactivity of phenoxyl radicals is mainly determined by the substituents in the 2-, 4- and 6-positions around the phenolic ring, because they undergo radical-radical coupling reactions. Therefore, if bulky substituents are placed in the 2-, 4- and 6-positions, it prevents the radicals from combining together. Figure 2a shows the CV of 2,4,6–tri–*tert*–butylphenolate that was produced by the addition of base to the phenolic starting material, where the CV is completely chemically reversible at a slow scan rate of 0.1 V s^−1^ due to the bulky *tert*–butyl groups preventing the formed phenoxyl radical from dimerizing. It has been argued that one of the reasons that α–TOH is favored biologically (for the antioxidant mechanism) is that the α–TO^•^ radical that forms via hydrogen atom transfer is particularly long-lived and able to take part in other radical termination reactions [18]. However, the CV data shown in Figure 2a,b indicate that α–TO^•^ is not especially long-lived compared to other bulky phenoxyl radicals.

The blue dashed trace in Figure 2b is the voltametric scan that is extended past the first oxidation peak of α–TO^−^ to form α–TO^•^, to further oxidize the phenoxyl radical to the diamagnetic cation, α–TO^+^. The second electron transfer at approximately 0.2–0.4 V vs. Fc/Fc^+^ is close in oxidative current magnitude (*i*_p_^ox^) to the first one-electron transfer peak at approximately −0.9 V vs. Fc/Fc^+^ due to both processes involving one-electron. The process at 0.2–0.4 V vs. Fc/Fc^+^ shows evidence of chemical reversibility in the sense that there is a reductive peak (*i*_p_^red^) when the scan direction is reversed, suggesting that α–TO^+^ can be reduced back to α–TO^•^. However, there appears to be two oxidative peaks observed between 0.2–0.4 V vs. Fc/Fc^+^ during the forward scan suggesting more complicated electrochemical behavior. It has been suggested that the extra peaks may be from reaction products of the initially formed α–TO^•^ since it takes several seconds to scan from −0.9 V vs. Fc/Fc^+^ to 0.2–0.4 V vs. Fc/Fc^+^, giving time for other homogeneous reactions to occur, or alternatively, adsorption effects may also be responsible for the extra peaks [54].

When a strong organo-soluble acid is added to the solvent such as CF_3_SO_3_H or CF_3_COOH, the voltammetry changes to that shown in the red trace in Figure 2d. In this instance the scan needs to be commenced at ≥0 V vs. Fc/Fc^+^ to avoid reduction of the acid. It can be seen in the red trace in Figure 2d that the first electron transfer process occurs at a similar potential to what occurs under approximately neutral conditions [c.f. green trace in Figure 2c]. However, the *i*_p_^ox^ is significantly less under acidic conditions than in the absence of acid. This is because under strong acidic conditions, the equilibrium reaction of the α–TOH^+•^ cation radical dissociating into α–TO^•^ favors the back reaction, thus the α–TOH^+•^ can survive at the electrode surface and in the bulk solution for at least 30 min and has been characterized by EPR and UV-vis spectroscopies [52,54,56]. Because α–TOH^+•^ has a significant lifetime in acidic conditions, it can be further oxidized when the potential is extended in the positive direction. Thus, the process that is observed at approximately +1.4 V vs. Fc/Fc^+^ in the red trace in Figure 2d is due to the one-electron oxidation of α–TOH^+•^ to the dication, α–TOH^2+^, as shown by the red pathway in Scheme 4. However, although the voltammetry suggests that α–TOH^2+^ is formed, controlled potential electrolysis and spectroscopic experiments indicate that the α–TOH^2+^ rapidly deprotonates into the diamagnetic cation, α–TO^+^ even in the strong acidic environment [54,56]. Therefore, all pathways in acid, base and under approximately neutral conditions (pure aprotic organic solvents) lead to the formation of α–TO^+^ as a stable product (in the absence of water). α–TO^+^ can be reduced back to the starting material under acidic conditions, but electrolysis experiments have indicated that it occurs via reduction to α–TO^•^, followed by protonation to form α–TOH^+•^, then further reduction to α–TOH (as shown in the reverse red pathway in Scheme 4) [54]. It is also noteworthy that the long lifetimes of the cation radicals of the tocopherols and tocotrienols are not observed for other phenols, whose cation radicals are much more acidic and therefore do not survive in solution for appreciable lengths of time [54,56].

#### 2.2.4. Kinetic Measurements and Effects of Methyl-Substitution from Different Tocopherols/Tocotrienols

There have been several detailed electrochemical studies aimed at determining the kinetic and thermodynamic parameters associated with the electrochemical reactions in Scheme 4 as well as the hydrolysis reactions shown in Scheme 2 for all of the tocopherols [56,60,65]. These studies were performed in the aprotic organic solvents acetonitrile, dichloromethane, dimethylsulfoxide and dimethylformamide, mainly because these solvents have reasonably high dielectric constants that enable electrochemistry to be performed in the presence of suitable chemically inert supporting electrolytes such as tetrabutylammonium hexafluorophosphate (Bu_4_NPF_6_) or tetrabutylammonium tetrafluoroborate (Bu_4_NBF_4_). It has been established that the length of the phytyl chain has no effect on the electrochemical properties of vitamin E (provided it remains as a simple alkyl group) [54,55], thus it would be expected that the tocopherols and tocotrienols behave in the same way electrochemically, although to date only detailed voltammetry experiments have been performed on the tocopherols. Nevertheless, it was established that the voltammetry was affected by the degree of methylation about the phenolic ring, hence the particular isomer (α, β, γ or δ) display varying voltammetric features.

The first study that compared all of the tocopherols together found that they did all undergo the same general reaction mechanism as shown in Scheme 2 and Scheme 4, but the biggest difference was the chemical reversible nature of the voltammetric response under neutral conditions [56]. For example, it was found that in the aprotic solvents CH_3_CN or CH_2_Cl_2_, α–TOH and β–TOH displayed chemically reversible voltammetry at all scan rates, whilst γ–TOH and δ–TOH were only partially chemically reversible at slow scan rates [56]. The implication to this result was that the diamagnetic cations (TO^+^) that are formed by –2e^−^/–H^+^ oxidation are more long-lived in solution for the α– and β–isomers, compared to the δ– and γ–isomers. Electrochemical experiments performed in the presence of strong acid indicated that the cation radicals (TOH^+•^) of all the tocopherol isomers were relatively long-lived in solution and EPR and UV-vis spectra could be obtained [56].

More detailed voltammetry experiments were conducted for the model versions of the tocopherol isomers where the phytyl tails were replaced with methyl groups and the electrochemical data were modelled using digital simulation techniques to obtain values for the homogeneous chemical steps occurring in Scheme 2 and Scheme 4 [60]. One very important reaction is the proton transfer reaction that occurs between TOH^+•^ and TO^•^ (Equation (3)) because the forward and reverse reactions must be fast and completely chemically reversible to account for how all of the tocopherols can be reversibly converted into their diamagnetic cations at fast voltametric scan rates.

TOH^+•^ ⇌ TO^•^ + H^+^ (3)

*K*_eq_ = [TO^•^][H^+^]/[TOH^+•^] (4)

TO^•^ + TO^•^ → non-radical products (5)

Digital simulations allowed estimates of the forward deprotonation rate constants between 10^3^–10^5^ s^−1^ (depending on the exact tocopherol), the reverse protonation rate constants between 10^6^–10^10^ L mol^−1^ s^−1^ (essentially diffusion controlled) and the corresponding equilibrium constants << 1 (~10^−4^–10^−5^) (Equation (4)). The very fast rate constants for the reverse reactions are the reason for the chemical reversibility going from TOH to TO^+^ (via –2e^−^/–1H^+^) because the intermediate TO^•^ radicals are reactive, hence if they were not quickly protonated during the reverse reduction reaction, they would undergo other self-bimolecular reactions (Equation (5)). Furthermore, although the equilibrium constant of 10^−4^–10^−5^ for Equation (4) seems extremely low and therefore, strongly favors the back reaction, when it is considered that that the forward reaction is first order and the backward reaction is second order, the concentration of the species at the electrode surface can be calculated to be similar. The low equilibrium constant for Equation (4) is consistent with the tocopherol cation radicals being relatively non-acidic, which is highly unusual for phenolic compounds whose corresponding cation radicals are highly acidic [58].

The measurements described in the previous paragraphs were mainly performed in CH_3_CN or CH_2_Cl_2_ [54,55,56,57,59,60,61,63,64,65,67]. The aprotic solvents DMF and DMSO are often considered to have similar properties to CH_3_CN, but it was found that the oxidized compounds were significant less stable in DMF and DMSO compared to CH_3_CN and CH_2_Cl_2_ [60]. Furthermore, adding non-polar solvents such as hexane or toluene to CH_3_CN or CH_2_Cl_2_ resulted in the immediate chemical instability of the rapid loss of the diamagnetic cation, α–TO^+^, of vitamin E [55]. Therefore, the choice of solvent for improving the lifetime of the oxidized intermediates is critical, with CH_3_CN and CH_2_Cl_2_ being the optimal choices at this time.

Another factor that is critical in improving the lifetime of the oxidized species, especially the diamagnetic cations, TO^+^, is the trace water content of the solvent since they are known to undergo a hydrolysis reaction. To accurately determine the rate of the hydrolysis reaction of the diamagnetic cations with water, it is essential that the trace water content of the solvent is known. Nevertheless, it is very rare that the water content of organic solvents is accurately reported during voltammetry in non-aqueous solvents, partly because electrochemical cells and apparatus are extremely difficult to keep moisture free, hence the trace water content of the solvent continually changes of the course of measurements unless experiments are conducted in a laboratory glove box [81]. Furthermore, it has been reported that the trace water content of solvents can shift the potentials of electrochemical reactions due to strong hydrogen bonding interactions, especially observed during the reduction of quinones [82,83,84,85,86,87,88], although phenols are also shown to undergo shifts [65,86]. A convenient method has been established for drying solvents used for electrochemistry by placing the solvent, electrolyte and compound for analysis inside a vacuum syringe containing pre-dried 3Å molecular sieves and storing the solution for 24 h [81,84,85]. Karl Fischer coulometric titrations indicated that by using this method the water content of the CH_3_CN could be lowered to <1 × 10^−3^ M, which is less than the concentration of analyte typically used in voltametric experiments (1–10 × 10^−3^ M) [81].

Voltammetric experiments with corresponding digital simulations were subsequently carried out on (CH_3_)α–TOH in CH_3_CN in the presence of carefully controlled water contents in order to determine the rate constant for the hydrolysis reaction of (CH_3_)α–TO^+^ with H_2_O and measure how hydrogen bonding affected the electrode potentials [65]. It was found that as water (between 0.010 M to 6 M) was progressively added to the CH_3_CN, the oxidation peak of (CH_3_)α–TOH moved progressively towards more negative potentials due to hydrogen bonding between the phenolic hydrogen atom on (CH_3_)α–TOH and the oxygen atoms in the water molecules [65,87,88]. It was also found that the reductive (*i*_p_^red^) to oxidative (*i*_p_^ox^) peak current ratio (*i*_p_^red^/*i*_p_^ox^) for the oxidation process decreased as water was added to the solvent due to the (CH_3_)α–TO^+^ reacting irreversibly with H_2_O to ultimately form the quinone [(CH_3_)α–TOQ] (compound **10** in Scheme 2) and therefore, (CH_3_)α–TO^+^ not being able to be reduced back to the starting material. By varying the voltammetric scan rate and water content and modelling the data with digital simulation techniques, an estimate of the rate of reaction of (CH_3_)α–TO^+^ with H_2_O was made of 11 L mol^−1^ s^−1^ at 25 °C [65].

#### 2.2.5. Difference between ECE and Disproportionation Mechanisms

The electrochemical reactions shown in Scheme 4 under neutral conditions are both assumed to occur heterogeneously, that is, to/from the electrode to the compound within the diffusion layer at the surface of the electrode as in Equations (6) and (7), interspaced with the homogeneous proton transfer in Equation (3).

TOH ⇌ TOH^+•^ + e^−^ (6)

TO^•^ ⇌ TO^+^ + e^−^ (7)

TOH^+•^ + TO^•^ ⇌ TO^+^ + TOH (8)

Nevertheless, it is possible that the second electron transfer step can also occur homogeneously according to Equation (8), where a cation radial (TOH^+•^) oxidizes another neutral radial (TO^•^) to form the diamagnetic cation (TO^+^) and regenerate the starting material (TOH), which constitutes a disproportionation mechanism. The ECE mechanism (Equations (3), (6) and (7)) occurs by the same number of electrons (i.e., 2) as the disproportionation mechanism (Equations (3), (6) and (8)), because in the case of the disproportionation mechanism, the TOH that is regenerated according to Equation (8) will immediately undergo oxidation at the electrode surface to form another molecule of the cation radical. Therefore, because both mechanisms occur by the same number of electrons, it is exceedingly difficult to differentiate them by modelling the current-voltage data, especially where the voltammograms are affected by complex behavior such as adsorption or hydrogen bonding. Professor Amatore, who this Special Issue of *Molecules* is devoted to, performed pioneering work on differentiating ECE and disproportionation mechanisms using linear sweep voltammetry [89], although for the present case of vitamin E, the ECE or disproportionation mechanisms have not been differentiated. Costentin argued that a disproportionation reaction was more likely for α–TOH because of the low equilibrium constant and a moderately fast deprotonation reaction (Equation (3)) [90]. However, an argument in favor of the simpler ECE mechanism is that it requires only one homogeneous chemical reaction to be fast and completely chemically reversible (Equation (3)), while the disproportionation mechanism requires two chemical reactions to be fast and chemically reversible (Equations (3) and (8)).

#### 2.2.6. Diamagnetic Cation

Of all the intermediate compounds shown in Scheme 2, the one that is most unusual is the diamagnetic cation (compound **6**). The reason that this compound is particularly interesting is that its lifetime is much greater than other analogous phenolic based compounds, as long as the water content of the solvent is low. While all phenols are thought to undergo the initial two-electron, one-proton oxidation process, it is remarkably that the vitamin E class of compounds produce a stable diamagnetic cation (at least for the α–form), and that the reaction is chemically reversible, so that reduction of the diamagnetic cation results in the complete regeneration of the starting material. The only other report of a long-lived phenoxenium cation is 2,6–di–*tert*–butyl–4–(4–dimethylaminophenyl)phenoxenium, which has very bulky substituents in the 2, 4 and 6–positions [91,92,93].

**Scheme 5:**
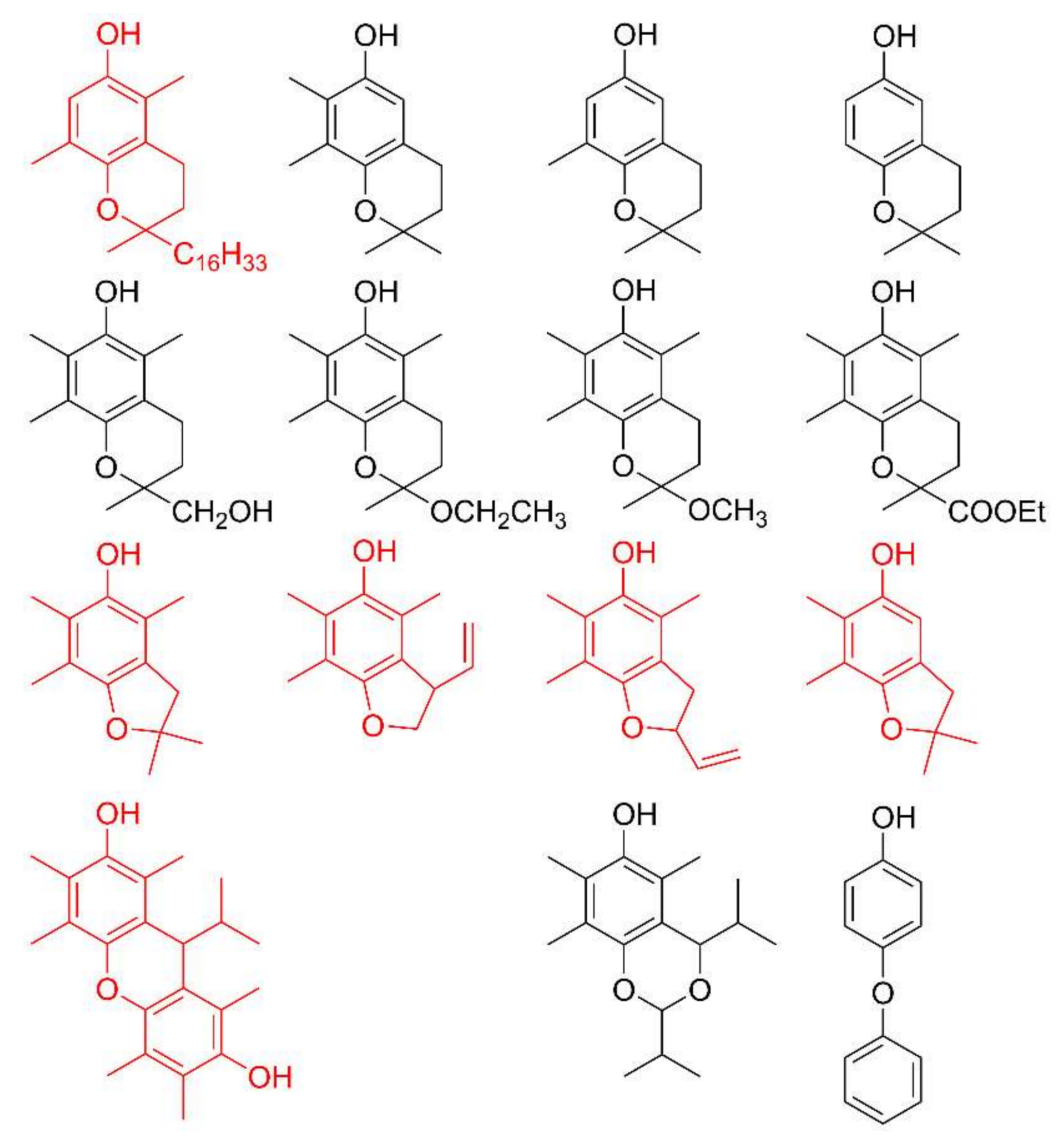
Structures of phenolic compounds for electrochemical oxidation experiments [56,59]. Compounds in red show clear evidence of forming semi-stable diamagnetic cations when oxidized in a –2e^−^/–1H^+^ process.

**Figure 3:**
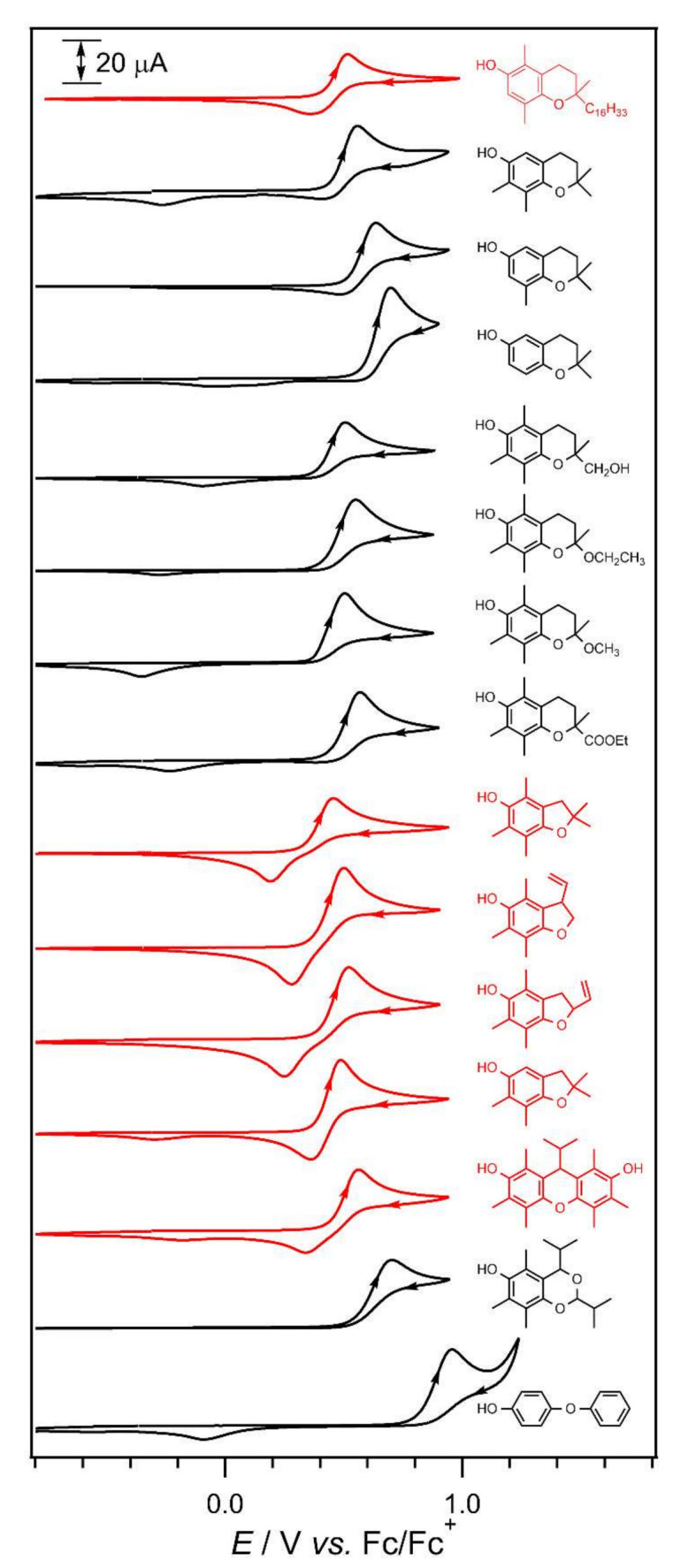
Cyclic voltammograms of 2.0 mM substrates in CH_3_CN with 0.2 M *n*-Bu_4_NPF_6_ recorded at a 1 mm diameter circular planar Pt electrode at a scan rate of 0.1 V s^−1^ at T = 293 K [56,59].

Having established that the high chemical stability of the diamagnetic cation derived from oxidation of the parent α–TOH was unusual compared to most other related compounds that typically have lifetimes in the microseconds [94,95,96,97,98,99], experiments were conducted to determine how much the structure could be changed whilst still allowing the oxidative production of a stable diamagnetic cation. For these experiments, a range of phenolic compounds were prepared with structures similar to α–TOH, but with different functional groups or atoms placed around the chroman-6-ol structure (Scheme 5) [59]. The compounds in Scheme 5 were all synthesized except for β–TOH, which is the natural compound and is drawn in the top left-hand corner (there is currently no published synthetic procedure for the model form of β–TOH where the phytyl chain is replaced with a methyl group) [60]. Evidence for the formation of a long-lived diamagnetic cation having a lifetime of at least few seconds came from cyclic voltammetry (CV) experiments. If a forward and reverse peak were detected during CV experiments at a scan rate of 0.1 V s^−1^ similar to that seen for α–TOH, then it was taken as confirmation of the existence of the diamagnetic cation that could be reduced back to the starting material (Figure 3) [56,59].

The molecules on the top row in Scheme 5 were assessed to determine the effect of methyl substitution of the phenolic ring. Only one compound showed evidence of a moderately stable diamagnetic cation upon electrochemical oxidation, which was the compound with two methyl groups opposite to each other (i.e., β–TOH). Therefore, it appears that the position of methyl substitution of the aromatic ring is critical to the lifetime of the diamagnetic cation.

The second row of molecules in Scheme 5 are based on α–TOH but have had the phytyl tail replaced with oxygenated functional groups including an alcohol, ethers and an ester. None of these compounds showed any evidence of chemical reversibility during CV experiments (Figure 3), suggesting that their diamagnetic cations were relatively short-lived. However, CV experiments are usually conducted between 1–10 mM of analyte, which is often below the level that trace water is present in organic solvents unless scrupulous care is taken keeping the solvent dry. Therefore, it is likely that the analyte concentration is less than the trace water concentration for the CVs shown in Figure 3. Subsequently, it was found that when the concentration of analyte was increased so it was much greater than the trace water content, some of the compounds in the second row in Scheme 5 showed evidence of forming semi-stable diamagnetic cations when they were chemically oxidized with NO^+^ [63].

The third row of compounds in Scheme 5 have the 6-membered ring replaced with a 5-membered ring. Interestingly, all of these compounds showed evidence of the existence of the diamagnetic cation that could be reversibly reduced back to the starting material. The final row of compounds in Scheme 5 had a more complicated 6-membered ring structure or a bulky phenol group bonded to the ether oxygen, with only one of these compounds showing evidence of the existence of the diamagnetic cation. In terms of maximizing the lifetime of the diamagnetic cations, the general observation was that a ring structure around the ether oxygen was important as well as having a fully methylated aromatic ring and having simple alkyl substituents. The study also examined the existence of other reaction products of the oxidation process including cation radicals, phenoxyl radicals, hydroxy *para*–quinone hemiketals, and *para*–quinones [59].

It is known that the major decomposition reaction of the diamagnetic cations is via hydrolysis with trace water (or other adventitious nucleophiles) to ultimately form the *para*–quinone through a ring cleavage reaction (compound **10** in Scheme 2). Consequently, the next set of experiments conducted were aimed at determining whether the structure of α–TOH could be modified to make it much more resistant to hydrolysis reactions of its associated diamagnetic cation. It was postulated that the initial reaction with water occurs in the phenolic ring at the carbon atom adjacent to the formed carbonyl group (i.e., compound **7** in Scheme 2). Therefore, by replacing the methyl group in α–TOH in the position closest to the ether oxygen with a bulky substituent, it was reasoned that the rate of hydrolysis could be slowed down.

**Scheme 6:**
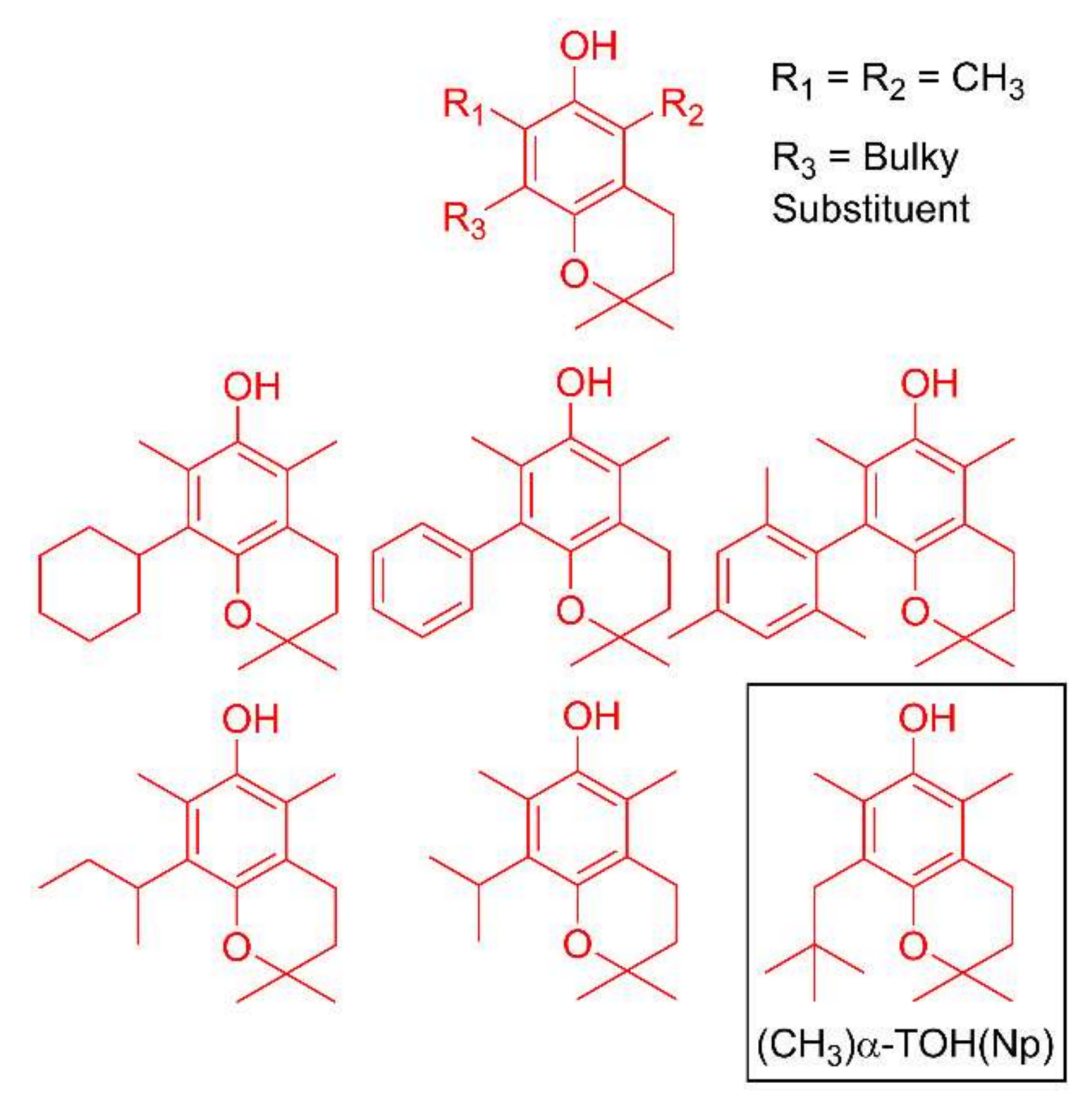
Structures of molecules based on α–TOH that were synthesized to offer improved resistance to hydrolysis reactions following oxidation [67].

Scheme 6 shows the series of molecules that were synthesized to test the hypothesis that reactions of their associated diamagnetic cations with H_2_O could be inhibited by placing bulky substituents in position R_3_ in the phenol ring [67]. The degree of inhibition of hydrolysis was experimentally determined by adding increasing amounts of water to acetonitrile solutions containing the compound and performing CV experiments to detect when the reverse reductive peak became smaller in current magnitude. The compounds in rows 2 and 3 in Scheme 6 were all found to produce relatively long-lived diamagnetic cations when oxidized, that were more resistant to hydrolysis than α–TOH, thereby confirming the hypothesis that having a bulky substituent in position R_3_ did retard hydrolysis of the formed diamagnetic cations. It was found that the α–TOH model derivative with the neopentyl substituent [abbreviated (CH_3_)α–TOH(Np)] formed a diamagnetic cation [(CH_3_)α–TO^+^(Np)] that was the most resistant to hydrolysis of the starting phenols shown in Scheme 6.

**Figure 4:**
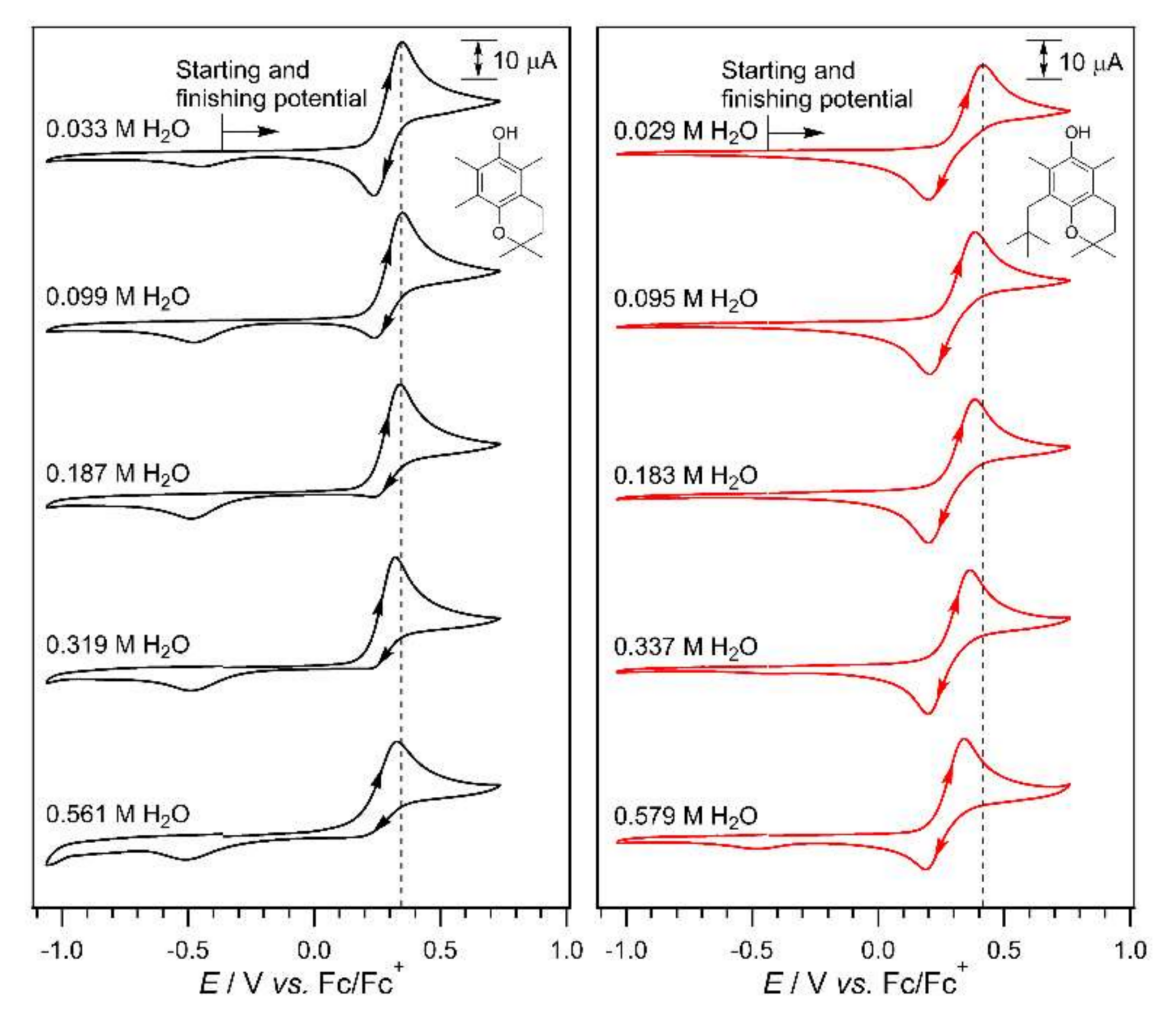
CVs of 2 mM of the α–tocopherol model compound [(CH_3_)α–TOH] and neopentyl derivative [(CH_3_)α–TOH(Np)] in CH_3_CN containing 0.2 M Bu_4_NPF_6_ at a 1 mm diameter circular planar Pt electrode at 22 ± 2 °C at a scan rate of 0.1 V s^−1^ at the specified concentration of water [67].

Figure 4 shows voltammograms of (CH_3_)α–TOH compared to (CH_3_)α–TOH(Np) when increasing amounts of water were added to CH_3_CN solutions. It can be observed that the apparent chemical reversibility of the voltammograms for the oxidation process of α–TOH at approximately 0.4–0.5 V vs. Fc/Fc^+^ is substantially decreased (i.e., *i*_p_^red^/*i*_p_^ox^ << 1) at water contents of 0.1 M, while at the same water concentration the *i*_p_^red^/*i*_p_^ox^-ratio observed for the CV of (CH_3_)α–TOH(Np) remains close to unity. It is only when the water content approaches 0.6 M that the *i*_p_^red^/*i*_p_^ox^-ratio begins to decrease from unity for CVs of (CH_3_)α–TOH(Np), indicating more resistance to hydrolysis reactions than α–TO^+^. The CVs of both (CH_3_)α–TOH and (CH_3_)α–TOH(Np) show the appearance of a new reductive process at approximately –0.5 V vs. Fc/Fc^+^ which is only evident on the reverse scan after first oxidizing the compounds, which increases in magnitude as more H_2_O is added (Figure 4). This new process at –0.5 V vs. Fc/Fc^+^ has been postulated to be associated with reduction of the hydroxy *para*–quinone hemiketal that forms via the hydrolysis reaction (i.e., compound **8** in Scheme 2) [59,60,63,65,67].

The reaction rate of H_2_O with the diamagnetic cation of (CH_3_)α–TOH(Np) was estimated by using digital simulation techniques to model variable scan rate CV data collected in acetonitrile containing varying amounts of water. The hydrolysis reaction of (CH_3_)α–TO^+^(Np) was estimated to be 0.5 L mol^−1^ s^−1^ [67], which is approximately 20 times slower than found for (CH_3_)α–TO^+^ [65].

#### 2.2.7. Quinone Methide

The *ortho*–quinone methide (compound **12** in Scheme 2) is also worthy of special attention as it has been shown to have an uncharacteristically long lifetime compared to related compounds, albeit in solution at very low temperatures. Rosenau et al. were able to generate the *ortho*–quinone methide of the (CH_3_)α–TOH model compound by chemical oxidation with Ag_2_O or Br_2_ that survived in CH_2_Cl_2_ at −78 °C for several seconds and reported that it could be converted back to (CH_3_)α–TOH under reducing conditions [100]. In Scheme 2 the formation of compound **12** is drawn as occurring through proton loss of the diamagnetic cation, but when using Ag_2_O or Br_2_ as oxidants, it is proposed to occur directly through proton loss from the methyl group in the phenoxyl radical [100]. It has also been reported that the *ortho*–quinone methide can exist in two forms that are electronically vastly different, with initially a zwitterionic intermediate formed through the chemical oxidation that converts into the *ortho*–quinone methide simply by a 90° bond rotation [101].

#### 2.2.8. Hydrolysis Products

**Figure 5:**
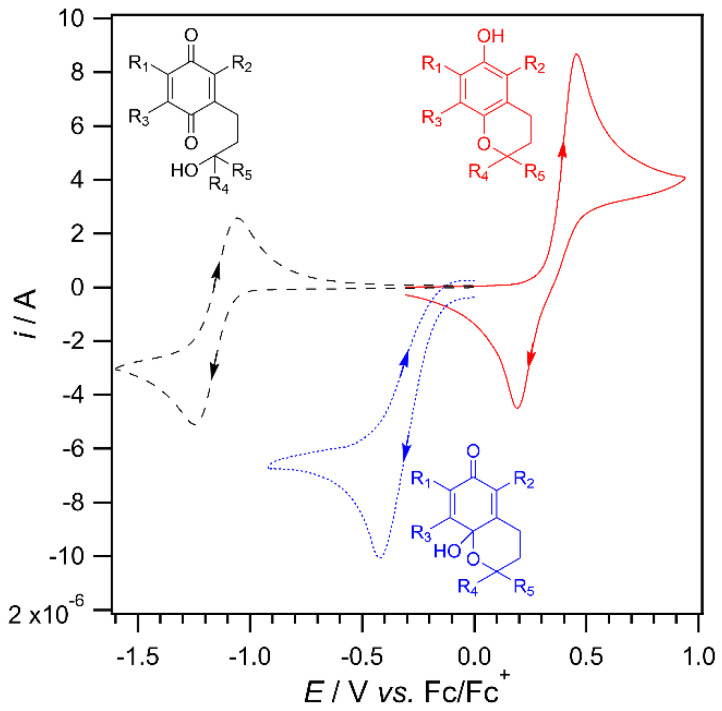
Representative voltammograms for oxidation of chroman-6-ol (red) and reduction of its associated hydroxy *para*–quinone hemiketal (blue) and *para*–quinone (black).

Cyclic voltammetry experiments are frequently conducted at low mM levels of analyte, typically 1–10 mM, in order to reduce the effects of uncompensated resistance and *IR* drop. Unless scrupulous care is taken in drying and handling organic solvents used for electrochemistry, the water content of the organic solvent is typically much greater than that of the analyte. Therefore, the long-term oxidation products of the vitamin E based compounds are often reported to be species formed by hydrolysis reactions with trace water. As shown in Scheme 2, the reaction of the diamagnetic cation (compound **6**) with water likely leads to intermediate compound **7**, which then reacts to form a hydroxy *para*–quinone hemiketal (compound **8**) before further conversion to the *para*–quinone (compound **10**). While *para*–quinones are well known stable species, *para*–quinone hemiketals related to the tocopherols are difficult to obtain in pure form and rapidly convert in the presence of acid to the quinones [59,60,63,65,102,103,104,105,106,107]. Voltammetry experiments have been used to provide evidence of the hydroxy *para*–quinone hemiketals of the tocopherol related compounds and they typically can be reduced at −0.2 to −0.6 V vs. Fc/Fc^+^, which is approximated 0.8 to 1.0 V less negative than the reduction potential of their corresponding quinones [59,63]. Figure 5 shows representative voltammograms of a starting phenol that is converted into a hydroxy *para*–hemiketal quinone and then into a *para*–quinone.

## 3. Spectroscopic Characterization of Each Class of Compound

The compounds in Scheme 2 can be divided into classes depending on their major functional groups and electronic state (neutral, open-shell, closed-shell, cations, anions, radicals, etc.), which impacts the methods chosen to use for characterization and the difficulty in obtaining good spectroscopic data, particularly for the compounds that have short lifetimes.

### 3.1. Tocopherols and Tocotrienols *(**1**)*

These compounds can broadly be classified as phenols based on the chroman-6-ol structure with variable methylation of the aromatic ring and with a saturated or unsaturated hydrocarbon tail. Since they are stable compounds of commercial importance, they have been extensively characterized using ^1^H and ^13^C NMR spectroscopy [108], mass spectrometry [109], vibrational spectroscopy [110], UV-vis spectroscopy [62], and chromatographic analysis [111], with much data available in spectroscopic databases [112] and literature reviews [113]. Most of the characterization was performed to aid in the quantification in complex matrixes such as foods and biological samples.

### 3.2. Phenolate Anions *(**2**)*

Phenolate anions are most easily prepared by reacting the phenolic starting materials with strong base [69,74,76]. The phenolates of the tocopherols can exist in solution for long periods but due to their very low oxidation potentials (~−0.9 V vs. Fc/Fc^+^) [74] undergo spontaneous reaction with O_2_ [114], therefore, need to be stored in in oxygen free environment. Preparation of the phenolates of tocopherols can be monitored using UV-vis spectroscopy where the λ_max_ shifts from 294 nm (for α–TOH) to 325 nm for the phenolate, α–TO^−^, in CH_3_CN (Table 1) [114]. In the presence of oxygen, α–TO^−^ was found to be oxidized to α–TO^•^ with the corresponding formation of the superoxide anion radical, (O_2_^−•^) [114]. Therefore, while α–TO^−^ can generate O_2_^−•^ (in the presence of O_2_), α–TOH is well known to be a scavenger (destroyer) of superoxide radicals via a hydrogen atom transfer mechanism [115,116].

### 3.3. Phenol Cation Radicals *(**3**)*

Phenol cation radicals are typically highly acidic species with very short lifetimes in solutions and are normally generated using photoionization and characterized with fast optical or vibration spectroscopic techniques [118,119]. Therefore, the phenol cation radicals derived from the tocopherols, or their model compounds are uncharacteristically long-lived in solution, which has enabled identification by electron paramagnetic resonance (EPR) spectroscopy, UV-vis spectroscopy and attenuated total reflectance (ATR)—Fourier transform infrared (FTIR) spectroscopy.

Experiments performed in the relatively low dielectric constant solvent, CH_2_Cl_2_, at low temperatures enabled EPR spectra of the cation radicals of all the tocopherols (α, β, γ, δ) to be obtained via one-electron chemical oxidation of the starting materials [120,121]. Using acidified CH_3_CN and CH_2_Cl_2_ shifts the equilibrium of the deprotonation reaction towards the protonated form so the one-electron oxidation of the starting materials leads to the formation of the cation radicals that survive in solution for at least several minutes at room temperature and enables detection by EPR spectroscopy [52,54,56].

Similarly, UV-vis spectra of the cation radicals of all the tocopherols can be obtained by in situ electrochemical oxidation of the starting materials in CH_3_CN with 0.1 M CF_3_SO_3_H [54] or CH_2_Cl_2_ with 1 M CF_3_COOH [52,56]. It has been found that even the titration of acid into organic solvents containing the tocopherols will lead to the spontaneous generation of the cation radicals [62]. α–TOH^+•^ displays a strong absorbance at 461 nm in acidified CH_2_Cl_2_ (Table 1) [62], with possibly another absorbance at 308 nm [56], although there remains uncertainty in the lower wavelength band due to possible interference from the presence of the diamagnetic cation. Pulse radiolysis experiments in hexane also led to the formation of α–TOH^+•^ and allowed the detection of its UV-vis spectrum [122]. The solutions of the cation radicals appear pale yellow at low micromolar concentrations and dark green at higher millimolar concentrations.

Measurements in acidified organic solvents also allowed the observation of solution phase infrared spectra of α–TOH^+•^ model compounds. An ATR-FTIR spectrum was obtained in CH_2_Cl_2_ containing 1 M CF_3_COOH of electrochemically generated (CH_3_)α–TOH^+•^, which had no absorbances detected in the carbonyl region (1500–1800 cm^−1^) [56]. A resonance Raman study on the water soluble derivative Trolox, (COOH)α–TOH^+•^, in aqueous acid solution led to the detection of a single strong band in the 1300–1800 cm^−1^ region at 1620 cm^−1^, which was assigned to a C=C ring stretching mode [123].

### 3.4. Phenoxyl Radicals *(**4**)*

α–TO^•^ is possibly the most widely studied intermediate of vitamin E because it is considered the primary compound formed in vitamin E’s proposed function as an antioxidant, via the starting material giving up a hydrogen atom to another free radical. However, unlike other phenoxyl radicals that have bulky substituents in the 2,4,6-positions and are very long-lived in solution [69,75], α–TO^•^ is reactive and does not survive long. The most common methods for generating α–TO^•^ from α–TOH is by pulse radiolysis or via a HAT reaction with a chemical agent, enabling its UV-vis spectrum to be obtained that shows a characteristic band at 425 nm with a shoulder at 408 nm (Table 1) [30,41,62,124]. Due to its paramagnetic state, α–TO^•^ has been studied by EPR spectroscopy with all the hyperfine coupling constants assigned [41,69,125,126]. Laser photolysis combined with probing within the long-wavelength absorption band of the radical at 425 nm enabled the time-resolved Raman spectra of the α–, β– and γ–tocopheroxyl radicals in methanol to be obtained [123].

### 3.5. Dications *(**5**)*

Evidence for the existence of the dications has come solely from CV experiments and there remains uncertainty as to whether they exist as discreet compounds or whether the electron transfer reaction between the monocation radical and dication occurs concertedly with a proton transfer to simultaneously produce the diamagnetic cation [diagonal PCET reaction between α–TOH^+•^ (compound **3**) and α–TO^+^ (compound **6**) in Scheme 2]. Therefore, no spectroscopic data is currently available.

### 3.6. Diamagnetic Cations (Phenoxeniums) *(**6**)*

Phenolic-derived diamagnetic cations are usually extremely reactive [95,96,97,98,99], but the diamagnetic cation of α–tocopherol was so long-lived that a version derived from its model methyl-substituted form [(CH_3_)α–TOH] was able to be isolated as a solid compound and crystallized with both non-nucleophilic [B(C_6_F_5_)_4_]^−^ and (CB_11_H_6_Br_6_)^−^ anions and their X-ray structures determined [57]. For the synthesis experiments, chemical oxidation using two equivalents of the one-electron oxidant NO^+^ (from NOSbF_6_) was used rather than electrochemical oxidation because it negated the need of supporting electrolyte [55,57]. Nevertheless, in order to isolate the (CH_3_)α–TO^+^, the experiments had to be performed at low temperatures (<−20 °C) and under very dry conditions to ensure hydrolysis reactions of the cation did not occur [55,57]. Although NO^+^ was proven to act as a very efficient oxidant of α–TOH and generate (CH_3_)α–TO^+^ in 100% yield [55], other phenols that are not fully substituted around the aromatic ring were found to undergo nitration reactions in the presence of NO^+^ [75,94].

**Figure 6:**
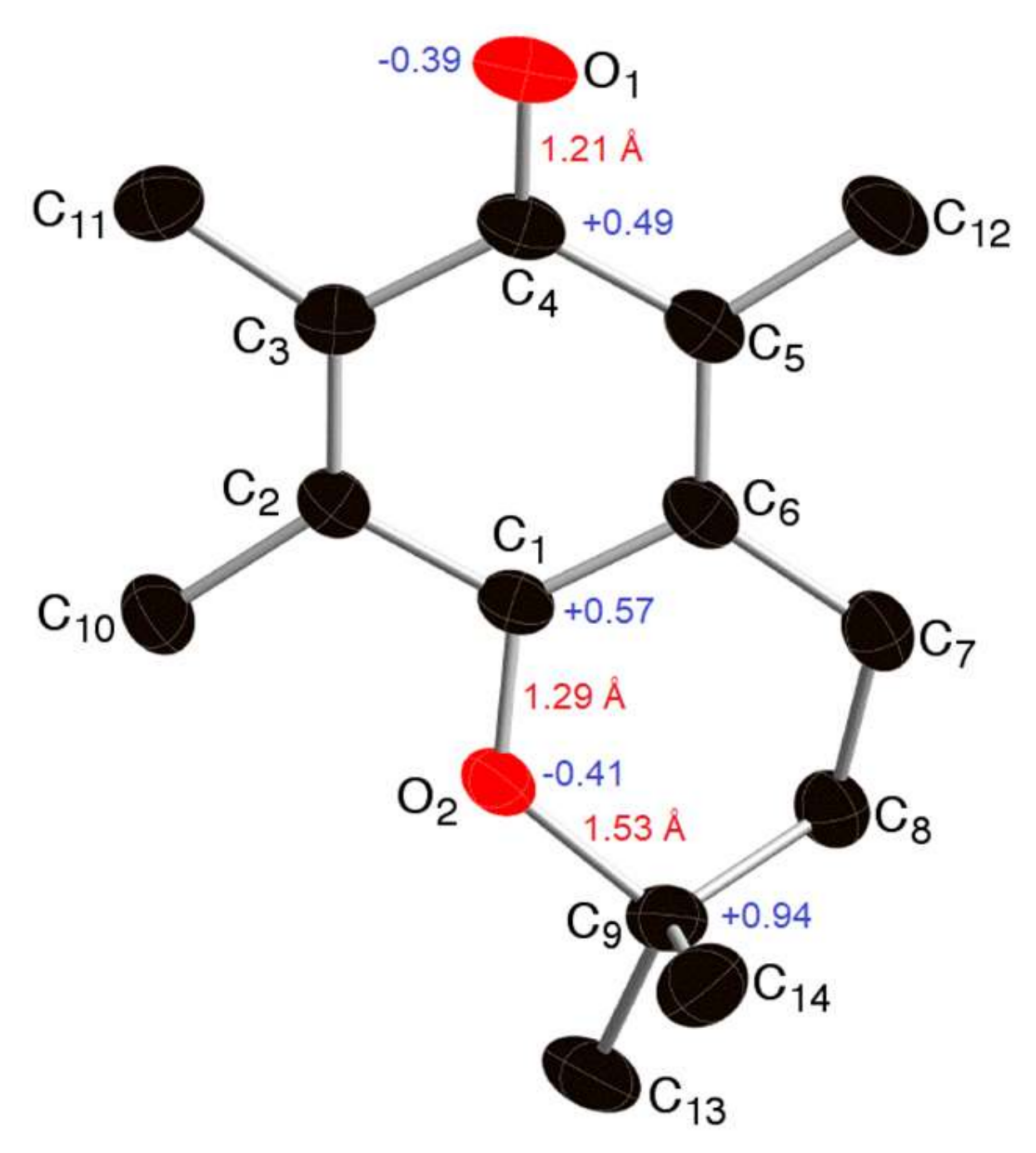
ORTEP plot for the molecular structure of (CH_3_)α–TO^+^ (crystallized with the [B(C_6_F_5_)_4_]^−^ counteranion) with bond lengths in given in red [57]. Thermal ellipsoids are drawn at the 50% probability level and hydrogen atoms are omitted. HF/6–31 + G* potential-derived electrostatic charges from DFT calculations are given in blue [55].

Figure 6 shows the ORTEP plot [57] for the molecular structure of (CH_3_)α–TO^+^ along with several highlighted bonds lengths (in red) and HF/6–31 + G* potential-derived electrostatic charges from DFT calculations (in blue) [55]. The C_9_–O_2_ bond length of 1.53 Å is particularly long for a carbon-oxygen single bond (ca. 1.4 Å), while in comparison, the C_1_–O_2_ bond length of 1.29 Å is shorter than expected for a C–O bond single. It appears that the chromanol ring structure is beneficial in stabilizing the C_9_–O_2_ bond despite it being long and therefore, weak. The C_4_–O_1_ bond length of 1.21 Å is consistent with what is expected for a carbonyl C=O double bond. DFT calculations indicated that the highest positive charge in the diamagnetic cation was surprisingly localized on the quarternary carbon (C_9_) with the oxygen atom (O_2_) having substantial negative charge characteristics [55]. These theoretical observations were supported by ^13^C NMR experiments that showed that the chemical shift of 73.1 ppm for the quaternary carbon in the neutral phenol starting material, shifted to 99.9 ppm in the diamagnetic cation, with the large downfield shift confirming that C_9_ was strongly deshielded in the cation [55].

Infrared spectra of the diamagnetic cations of the tocopherols [54,55,56] and several other related compounds shown in Scheme 5 [63] appear similar and display three strong bands between 1681 cm^−1^ and 1604 cm^−1^ (but with the highest wavenumber band often appearing as a shoulder on the most intense band). DFT calculations performed on (CH_3_)α–TO^+^ confirmed that the structure was similar to that of a quinone consisting of a carbonyl group and a cyclic diene [55] so the infrared spectra between ~1800–1500 cm^−1^ would expect to show at least 3 absorbances corresponding to an asymmetric diene ring stretch, a symmetric diene ring stretch, and a carbonyl stretch (although in each case other coupled vibrations contribute to the modes). For (CH_3_)α–TO^+^, DFT calculations predicted the absorbance at close to 1600 cm^−1^ was associated with the asymmetric ring stretching mode, while the two close bands that occur at 1663 cm^−1^ (which appears as a shoulder) and 1650 cm^−1^ were predicted to be due to the carbonyl stretch and symmetrical ring stretch, respectively [55]. This prediction implies that the carbonyl stretch is less intense than the symmetrical ring stretch. Therefore, there remains some uncertainty as to which is the carbonyl stretching band as it is usually considered to be the most intense band within the 1800–1500 cm^−1^ region. Nanosecond time resolved resonance Raman spectroscopy in CH_3_CN/H_2_O solutions of 4–acetoxy–4–aryl–2,5–cyclohexadienone led to the detection of a phenoxenium cation, which displayed an asymmetric diene ring stretch at 1525 cm^−1^, a symmetric diene ring stretch at 1593 cm^−1^, and a C=O stretch at 1635 cm^−1^ [97].

α–TO^+^ and its model compounds are colored bright orange and display strong UV-vis absorbances at 298 nm and 425 nm (Table 1) [54,55,56].

### 3.7. Hemiketals and Hemiketal Anions *(**8**,**9**)*

The synthesis of the hydroxy *para*–quinone hemiketal (compound **8** in Scheme 2) derived from the (CH_3_)α–TOH model compound has been reported by simply purging solutions of the starting material with oxygen gas [103,104]. The hemiketal was described as a pale-yellow oil, although it was difficult to completely purify as it readily converted back to the starting material or reacted to form other products, possibly involving an *ortho*–quinone methide intermediate [104]. The infrared spectrum showed a proposed C=O absorbance at 1650 cm^−1^ and a UV-vis absorbance was detected at λ_max_ = 239 nm in methanol (Table 1), and ^1^H NMR data were reported [104]. There are several other reports on the existence of *para*–quinone hemiketals related to the tocopherols where a methoxy group is present in place of the hydroxy group shown in compound **8** in Scheme 2, although these compounds are difficult to obtain in pure form and rapidly convert in the presence of acid to the *para*–quinones [105,106,107]. Hydroxy *para*–quinone hemiketals produced from the electrochemical oxidation of compounds shown in Scheme 5 show ATR-FTIR absorbances at close to 1650 cm^−1^ for the solution phase compounds [63], although there is uncertainty as to whether this is due to the carbonyl group or is a ring stretching mode due to the quinoid structure, similar to the diamagnetic cations. Voltammetry and electrolysis experiments indicated that the hydroxy *para*–quinone hemiketals can be reduced back to the starting materials by applying a potential more negative than ~−0.4 V vs. Fc/Fc^+^, presumably by causing the loss of the hydroxide anion [63]. In Scheme 2, the reduction reaction of the hydroxy *para*–quinone hemiketal is written as involving one electron to form an anion radical. However, the CV data collected for these compounds typically display chemically irreversible behavior (Figure 5) suggesting that the anion radical is not long-lived, and the reduction likely involves the transfer of more than one electron [59,63].

### 3.8. Quinones and Quinone Anions *(**10**,**11**)*

*Para*-quinones (Q) and their reduced forms (Q^−•^ and Q^2−^) are well known long-lived compounds with important biological functions (such as for vitamin K_1_ and coenzyme Q_10_) and interesting electrochemical properties [66,68,80]. UV-vis of the *para*–quinone of the model compound (CH_3_)α–TOQ showed overlapping UV-vis absorbances at 258 and 266 nm in CH_3_CN [63] and at 262 nm in CH_3_OH (Table 1) [104].

In aprotic solvents, quinones including α–TOQ and its model compound, (CH_3_)α–TOQ, have been shown to be reduced in two one-electron steps to form first the anion radicals [α–TOQ^–•^] (Equation (9)) and then at more negative potentials the dianions [α–TOQ^2−^] (Equation (10)) [48,49,50,59,127]. Recent electrochemical studies have shown that quinone anion radicals and especially the dianions undergo very strong hydrogen bonding interactions with water molecules, that can be present in aqueous solutions or in trace amounts in organic solvents [80,81,82,83,84,85,86,87,88,128]. In aprotic organic solvents, the hydrogen bonding interactions cause the second more negative electron transfer step to progressively move towards more positive potentials as water is added to the solution, so that at a high enough water content, the two one-electron processes merge into one two-electron chemically reversible process (Equation (11)). Therefore, in unbuffered aqueous solutions or in organic solvents containing sufficient water, quinones are always reduced in a two-electron process to form the hydrogen bonded dianion. Nevertheless, the anion radicals of quinones are known to exist in fully aqueous solutions, with the reason for this being a comproportionation equilibrium reaction where the dianion reacts with the starting material to form two molecules of the anion radical (Equation (12)) [128]. Therefore, due to this comproportionation reaction, UV-vis spectra of the Trolox derived anion radical, (COOH)α–TOQ^–•^ has been reported in aqueous solutions, with λ_max_ = 440 nm [117].

(9) $$Q+e^{-}⇌Q^{-•},\;E_{\;f\left(1\right)}^{0}\;/\;V$$

(10) $$Q^{-•}+e^{-}⇌Q^{2-},\;E_{\;f\left(2\right)}^{0}\;/\;V$$

(11) $$Q+2e^{-}+yH_{2}0⇌Q{\left(H_{2}O\right)}_{y}^{2-},\;E_{\;f\left(3\right)}^{0}\;/\;V$$

(12) $$Q{\left(H_{2}O\right)}_{y}^{2-}+Q\;⇌Q{\left(H_{2}O\right)}_{x}^{-•}+Q{\left(H_{2}O\right)}_{x}^{-•}$$

### 3.9. Quinone Methides *(**12**)*

Quinone methides are highly reactive species, so it is significant that one derived from oxidation of α–TOH can be stabilized long enough to be characterized by NMR spectroscopy [100,101]. Oxidation of α–TOH with Ag_2_O at −78 °C in CH_2_Cl_2_ leads to the formation of the spiro-dimer within approximately 10 s. However, oxidation in the presence of *N*-methylmorpholine-*N*-oxide slows down the reaction, so that the formation of the spiro-dimer took around 20 min for completion. ^1^H and ^13^C NMR studies at −78 °C indicated that the *N*-oxide stabilized *para*–quinone methide had a zwitterionic aromatic resonance structure with a positive charge on the methylene carbon and a negative charge on the former phenolic oxygen atom [100].

Table 2 provides a summary of literature reports giving spectroscopic characterization data of oxidized intermediate forms of α–TOH and its model compounds.

## 4. Conclusions

The electrochemical behavior of the α–, β–, γ–, δ–isomers of the tocopherols and tocotrienols collectively comprising vitamin E are broadly similar to what is observed during the oxidation of other phenolic compounds, but with some notable differences with respect to the lifetimes of several of the oxidized intermediates. In particular, the cation radicals of the α–, β–, γ–, δ–isomers formed by one-electron oxidation of the starting phenols are all uncharacteristically long-lived in acidic organic environments due to the phenolic hydrogen atoms being very weakly acidic and the acid dissociation constants favoring the protonation reaction (*K*_dissociation_ << 1), especially in non-aqueous solvents.

In CH_3_CN or CH_2_Cl_2_ and in the absence of added acid or base, the isomers comprising vitamin E are oxidized in a –2e^−^/–1H^+^ process to form diamagnetic cations, sometimes labelled as phenoxenium cations. The diamagnetic cations are reactive with even trace amounts of water and undergo hydrolysis reactions to firstly form hydroxy *para*–quinone hemiketals and then *para*–quinones as long-term products. The diamagnetic cation of the α–form (α–TO^+^) is the most resistant isomer to hydrolysis reactions and a model form where the phytyl chain was replaced by a methyl group [(CH_3_)α–TO^+^] has been isolated as a solid compound and its X-ray crystal structure determined. The long lifetime of α–TO^+^/(CH_3_)α–TO^+^ has been rationalized by it having a fully methylated quinoid ring which slows down H_2_O molecules reacting by providing steric hindrance, along with the chromanol ring structure enabling the positive charge to be delocalized away from the phenolic group. The long lifetime of α–TO^+^ is particularly noteworthy because the phenoxenium cations derived from other phenols usually only exist as transitory species that can be detected during ultrafast time-resolved spectroscopic measurements.

Because of the uniqueness of the long lifetime of α–TO^+^ compared to the other forms (β, γ and δ) as well as to other phenols, studies were performed to determine how much the structure could be varied whilst still enabling the diamagnetic cation to survive in organic solutions for > seconds in the absence of water. Since hydrolysis reactions are the main mode of reactivity of the diamagnetic cation, experiments were also performed to see if modifying the structure of α–tocopherol could lead to diamagnetic cations that were even more resistant to hydrolysis reactions. It was found that a version of α–TOH where one of the methyl groups in the phenolic ring was replaced with a bulkier neopentyl group led to a diamagnetic cation upon oxidation that was approximately 20 times more resistant to hydrolysis, based on kinetic measurements.

To date, most of the attention on oxidized forms of vitamin E has focused on the phenoxyl radical, partly because of its perceived role in vitamin E’s proposed role as an antioxidant, and partly because there are tools in place that enable it to be generated and studied in cells under in vitro conditions. However, the other oxidized forms of vitamin E, such as those shown in Scheme 2, may also exist in biological systems but they are more difficult to generate in a controlled way, and are therefore, more difficult to detect, especially if they participate in fast chemically reversible reactions. The purpose of this review was to highlight all of the oxidized forms of vitamin E with the intent that they could be looked for in future experiments under in vivo conditions. From a chemical perspective, α–TO^+^ is the most intriguing, since other compounds of its type are usually highly reactive. No biological functions have currently been linked to α–TO^+^, but it could potentially act as a signaling molecule through chemically reversible transformations. For example, interactions between α–TO^+^ and β–carotene have been shown to regenerate α–TOH and produce β–carotene^2+^ [64].

One area of research that requires considerably more attention to fully understand the biological properties of vitamin E and possibly its oxidized forms, is the exact location that the compound resides within the lipid bilayers. Existing experiments that have been used to probe vitamin E’s position within membranes have required the use of fluorescent probes attached to the structure and/or model membranes, which will likely affect its exact orientation to a greater of lesser extent [31,32,33,129,130,131]. It is significant that the α–TTP specifically targets the *RRR*–chiral form, which is likely because of the exact geometry that is adopts. Therefore, being able to conduct in situ spectroscopic experiments on *RRR*–α–tocopherol in real mammalian cells is an essential goal to strive for to enable an optimal understanding of its biological functions and mechanism of action.

## Figures and Tables

**Table 1:** Selection of solution phase UV-vis absorption data for vitamin E and its model compounds and their oxidized forms.

Compound	Solvent	λ_max_/nm	Literature
α–TOH	hexane	298	[62]
	CH_3_CN	294	[54]
(CH_3_)α–TOH	CH_3_CN	294	[55]
(CH_3_)α–TO^−^	CH_3_CN	325	[114]
(CH_3_)α–TOH^+•^	CH_2_Cl_2_/CF_3_COOH	461	[62]
	CH_2_Cl_2_/CF_3_COOH	308,465	[56]
α–TOH^+•^	CH_3_CN/CF_3_SO_3_H	301, 464	[54]
α–TO^•^	chlorobenzene	424	[41]
(CH_3_)α–TO^•^	CH_2_Cl_2_	425	[62]
α–TO^+^	CH_3_CN	298, 425	[54]
(CH_3_)α–ΤO^+^	CH_3_CN	298, 425	[55]
(CH_3_)α–TOQ(OH)	CH_3_OH	239	[104]
(CH_3_)α–TOQ	CH_3_CN	258, 266	[63]
(COOH)α–TOQ^−^^•^	H_2_O	440	[117]

**Table 2:** Selection of literature reports of spectroscopic data on oxidized forms of vitamin E or is model compounds *^a^*.

Compound *^b^*	UV-Vis and Pulse Radiolysis	Infrared and Raman	EPR	NMR	X-ray Crystallography
	Literature Numbers from Reference Section				
TO^−^ (**2**)	[114]				
TOH^+•^ (**3**)	[52,54,56,62,122]	[56,123]	[52,54,56,120,121]		
TO^•^ (**4**)	[30,41,62,124]	[123]	[41,69,125,126]		
TO^+^ (**6**)	[54,55,56]	[54,55,56,63]		[55]	[57]
TOQ(OH) (**8**)	[104]	[63,104]		[104]	
TOQ (**10**)	[63,104]	[63,104]		[104]	
TOQ^−•^(**11**)	[117]				
QM (**12**)				[100,101]	

*^a^* The reference may refer to data from any of the tocopherols (α, β, γ, δ). *^b^* The general structure of the intermediate is given by the α–TOH derived form (or its model compounds) labelled in Arabic numbers in Scheme 2.

## Data Availability

Not applicable.
